# Thermoresponsive Platinum(II) 2,6-Di(pyrid-2-yl)pyrazine Complexes with Unusual Aggregation Behavior upon Heating

**DOI:** 10.1021/jacs.5c07413

**Published:** 2025-07-04

**Authors:** Tracy Ho-Ying Chan, Ziyong Chen, Ming-Yi Leung, Michael Ho-Yeung Chan, Eric Ka-Ho Wong, Wai Kit Tang, Vivian Wing-Wah Yam

**Affiliations:** Institute of Molecular Functional Materials and Department of Chemistry, 25809 The University of Hong Kong, Pokfulam Road, Hong Kong, P. R. China

## Abstract

A series of alkynylplatinum­(II) complexes with 2,6-di­(pyrid-2-yl)­pyrazine ligand has been synthesized and characterized. Interestingly, the complexes have been found to exhibit unprecedented thermochromic behavior with absorption maxima shifting to the longer-wavelength region upon increasing temperature. This phenomenon is significantly different from that in conventional platinum­(II) systems, where an elevated temperature typically promotes the dissociation of aggregates and diminishes MMLCT absorptions. Transmission electron microscopy (TEM) and variable-temperature ^1^H NMR spectroscopy suggest a thermoresponsive morphological transformation behavior from rods to circular morphologies, driven by the preferential formation of π–π and Pt···Pt interactions over hydrogen bonding interactions upon the disruption of hydrogen bonding at high temperature, as supported by solution-state Fourier transform infrared (FT-IR) spectroscopy together with computational studies. This unusual optical and morphological transformation is rarely observed, as it represents a stark contrast to the deaggregation behavior upon increasing temperature typical of the control and commonly found platinum­(II) complexes and organic compounds.

## Graphical abstract

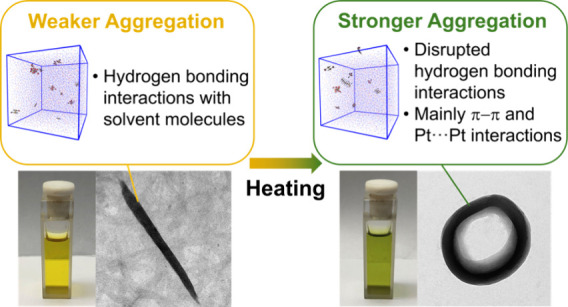

## Introduction

Over the past few decades, square-planar platinum­(II) complexes with a d^8^ electronic configuration have attracted tremendous attention owing to their rich photophysical and intriguing self-assembly properties due to the presence of non-covalent Pt···Pt and/or π–π stacking interactions.^1–15^ Previous systematic studies on alkynylplatinum­(II) terpyridine complexes have demonstrated intriguing solution-state self-assembly properties of this class of complexes.^16–23^ The self-assembly and spectroscopic properties of this class of complexes are found to be tuned by solvent composition,^16–18^ counter-anions,^19,20^ pH,^18,21^ and temperature.^22,23^ An interesting example is the polyhedral oligomeric silsesquioxanes (POSS)-functionalized alkynylplatinum­(II) terpyridine complex which exhibits a morphological transformation governed by the interplay of hydrophobic, Pt···Pt, and π–π stacking interactions by varying the solvent composition.^17^ In these previously reported studies, typical platinum­(II) complexes generally demonstrate deaggregation behavior upon heating.^18,22,23^

Hydrogen bonding is one of the most important classes of non-covalent interactions due to its high selectivity and directionality. In view of this, numerous supramolecular architectures and polymers have been developed with the aid of hydrogen bonds.^24–27^ It also plays a crucial role in nature as it provides structural stability to biomolecules such as the double-helical deoxyribonucleic acids^28^ and proteins.^29,30^ It is noteworthy that these biomolecules undergo denaturation due to the disruption of hydrogen bonding between the nucleic acids and amino acids, respectively, upon increasing temperature.^31–33^ In light of its nature, hydrogen bonding is a type of non-covalent interaction that may be utilized in the self-assembly of organic compounds and transition-metal complexes due to its selectivity and temperature-controllable formation and disruption. Despite the growing interest in the development of self-assembly of platinum­(II) complexes, most efforts have been directed toward the utilization of various non-covalent interactions including Pt­(II)···Pt­(II), π–π stacking, electrostatic and hydrophobic interactions between the platinum complexes as the driving force for self-assembly.^4–8,10,11,15–18,22,23,34–46^ While there has also been exploration of the contribution of hydrogen bonding in the assembly properties,^20,23,47–52^ the utilization of hydrogen bonding between the complex and the solvent molecules is relatively rare. It is anticipated that hydrogen bonding interactions between the platinum­(II) complexes and the solvent molecules could result in interesting aggregation behaviors, which could possibly be modulated by temperature.

In this context, a class of alkynylplatinum­(II) complexes with a tridentate *N*-donor pincer ligand that includes a pyrazine moiety as a hydrogen bond acceptor (Scheme 1) and their drastic spectroscopic changes upon increasing temperature is reported. Such changes are found to be associated with the morphological transformation of the platinum­(II) complexes, which has not been demonstrated in typical alkynylplatinum­(II) polypyridine complexes with the exception of triblock copolymer-containing platinum­(II) terpyridine complexes.^53^ Unlike conventional platinum­(II) complexes that generally show deaggregation behavior upon heating, this study reports the unprecedented thermally induced enhanced aggregation behavior of small-molecule platinum­(II) complexes, which has not been demonstrated so far. Comparison studies with control complexes and solvents incapable of hydrogen bonding, together with computational and molecular dynamics (MD) studies, have led to the elucidation of the mechanism. It is envisaged that this work could pave the way for developing unique thermochromic materials through the controlled self-assembly of platinum­(II) complexes.

**Scheme 1:**
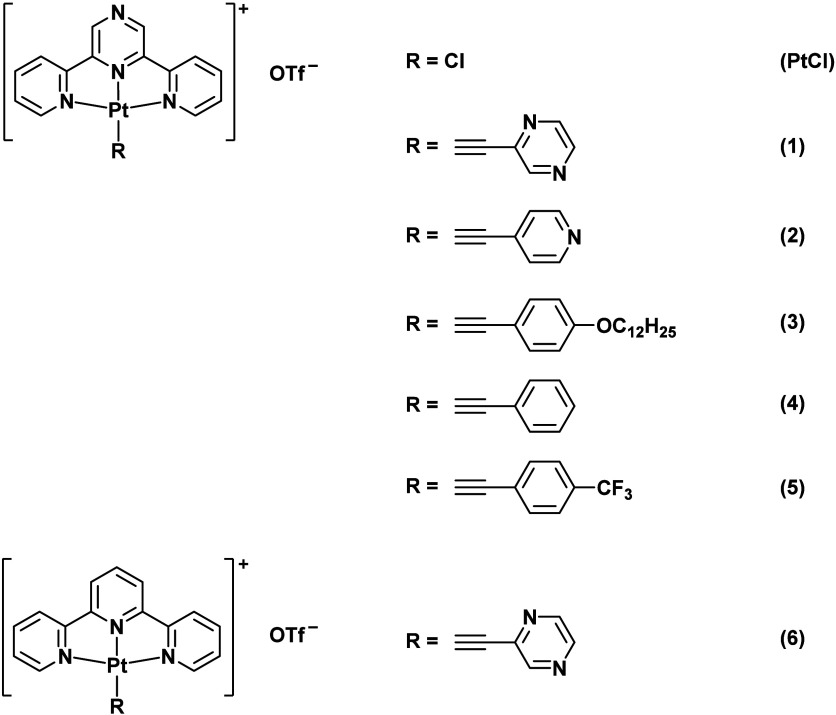
Structures of **PtCl** and Complexes **1**–**6**

## Results and Discussion

The platinum­(II) complexes were synthesized by reacting the chloroplatinum­(II) 2,6-(dipyrid-2-yl)­pyrazine (**PtCl**) or chloroplatinum­(II) terpyridine (tpy) precursor complexes^54^ with the corresponding alkynes in the presence of an excess of potassium fluoride in methanol. Subsequent purification by recrystallization of the crude product by slow diffusion of diethyl ether into concentrated acetonitrile solutions gave the complexes as red, green, brown, or purple solids. **1**–**6** have been characterized by ^1^H NMR spectroscopy and high-resolution ESI mass spectrometry.

Interestingly, two forms of single crystals for X-ray crystallography, including the red form and yellow form of **PtCl**, have been obtained by the slow diffusion of diethyl ether vapor into an acetonitrile and dilute acetone solution of the complex, respectively. The platinum­(II) metal centers in both the red and yellow forms of **PtCl** are found to adopt a distorted square-planar geometry due to the restricted bite angle of 2,6-(dipyrid-2-yl)­pyrazine (Figure S1a), which is also found in other structurally related platinum­(II) complexes with π-conjugated ligands.^21^ The red form of the precursor complex shows a dimeric structure stacking in a head-to-tail configuration with alternating long and short Pt···Pt distances of 3.523 and 3.285 Å (Figure S1b), indicating the presence of Pt···Pt interactions.^16^ The yellow form, on the other hand, exhibits a more zigzag arrangement with the platinum atoms equally spaced at 3.617 Å, suggesting a lack of intermolecular Pt···Pt interactions (Figure S1c).

Single crystals of **2** and **5** have also been obtained upon slow diffusion of diethyl ether into an acetonitrile solution of the complex. Similar to **PtCl**, two forms of single crystals of **2** are observed, but only the crystal structure of the red form could be obtained. Analogous to the chloro precursor complex, the platinum­(II) metal centers of **2** and **5** are in a distorted square-planar geometry (Figures S2a and S3a). The complex cations are found to stack dimerically in a head-to-tail configuration, as indicated by a C–Pt–Pt–C torsional angle of 180°. While the Pt···Pt distance is found to be 3.270 Å within the dimeric structure of **2**, suggesting the presence of significant Pt···Pt interactions (Figure S2b), Pt···Pt interactions are absent in **5** as indicated by the long Pt···Pt distances of 4.228 and 4.244 Å (Figure S3b).

Dissolution of **1**–**6** in pure acetonitrile is found to give yellow to pink solutions, and the corresponding UV–vis absorption spectra are depicted in Figure S4. The high-energy absorption bands at 300–360 nm are assigned as intraligand (IL) [π → π*] transitions of the alkynyl and the 2,6-(dipyrid-2-yl)­pyrazine ligands. The low-energy absorption bands at 400–600 nm are assigned as metal-to-ligand charge transfer (MLCT) [dπ­(Pt) → π*­(2,6-(dipyrid-2-yl)­pyrazine)] transitions mixed with ligand-to-ligand charge transfer (LLCT) [π­(alkynyl) → π*­(2,6-(dipyrid-2-yl)­pyrazine)] character.^16^ In general, these absorption bands are found to occur at lower energies than those of the corresponding terpyridine counterparts with the same alkynyl ligand. For instance, the MLCT/LLCT absorption bands of **1** (421 nm) and **4** (460 nm) occur at a longer wavelength than their tpy analogues, **6** (407 nm) and **[Pt­(tpy)­(C**≡**CC**_**6**_ **H**_**5**_ **)]­OTf** (**Ref)** (440 nm), respectively (Figure S4). Such a difference in the absorption energies is attributed to the stronger electron-accepting ability of 2,6-(dipyrid-2-yl)­pyrazine compared to that of terpyridine. This results in a more stabilized π*­(2,6-(dipyrid-2-yl)­pyrazine) orbital than the π*­(tpy) orbital, eventually giving rise to a lower MLCT/LLCT absorption energy. Moreover, these bands are also found to be sensitive to the nature of the alkynyl ligands. A blue shift in the MLCT/LLCT absorption wavelengths from **3** (511 nm) to **4** (460 nm) to **5** (439 nm) to **2** (429 nm) and to **1** (421 nm) has been observed (Figure S4). This could be attributed to the poorer electron-donating effect of the alkynyl ligands due to the presence of the more electronegative nitrogen-containing heterocycles or the electron-withdrawing −CF_3_ substituent when comparing pyrazine, pyridine, and trifluoromethylphenyl to phenyl or the even more electron-donating alkoxy-substituted phenyl rings. As a result, the π­(alkynyl) and the dπ­(Pt) orbitals will be stabilized, giving rise to a higher MLCT/LLCT absorption energy. Temperature-dependent UV–vis absorption studies of **1** in acetonitrile has been performed to investigate the character of the lowest-energy absorption band. Interestingly, a drop in intensity and a blue shift of the low-energy absorption band at *ca*. 590 nm has been observed with increasing temperature (Figure S5). Moreover, this low-energy absorption band at ca. 590 nm upon increasing concentration from 33 to 1380 μM is found to deviate from Beer’s law (Figure S6), indicating the intermolecular ground-state association of **1** via Pt···Pt interactions. This, together with the selected-area electron diffraction pattern showing two arcs with a distance of 0.34 nm (*vide infra*), suggests the low-energy absorption to be of MMLCT origin due to the assembly of the complex. Thus, the drop in intensity and the blue shift of the absorption band at *ca*. 590 nm suggest the possible disruption of metal–metal and/or π–π stacking interactions at elevated temperature. Interestingly, upon increasing diethyl ether content in the acetonitrile solution of **1**, there is a drop in the MLCT/LLCT absorption band at 430 nm with the concomitant growth of a lower-energy absorption band at *ca*. 590 nm, indicating the possible formation of aggregates via metal–metal and/or π–π stacking interactions induced by reduced solvation (Figure S7).

The self-assembly properties of the complexes in mixed solvent systems involving other polar protic solvents have been further studied. Increasing the methanol content in the acetonitrile solution of **1** gives no significant changes in the lowest-energy absorption band (Figure S8). Surprisingly, a drastic color change in such a mixed-solvent system upon heating is found, in addition to the solvent-induced aggregation. It is found that **1** dissolves in an acetonitrile–alcohol mixture to give a yellow solution, which changes dramatically from yellow to green upon increasing temperature and returns to yellow upon cooling (Figure 1). UV–Vis absorption studies of **1** in an acetonitrile–ethanol mixture (4:1 v/v) reveal the spectral changes responsible for the drastic color changes, in which a growth in a new absorption band at 620 nm, tentatively assigned to the MMLCT transition, concomitant with a drop in the absorption band at 420 nm with an isosbestic point at 460 nm upon increasing temperature, is observed (Figure 2). The MMLCT nature of the lowest-energy absorption band is further confirmed by the concentration-dependent studies of **1** in an acetonitrile–methanol mixture (4:1 v/v), in which the absorption band at 590 nm in the concentration range of 3–1210 μM is found to deviate from Beer’s law (Figure S9). This is in marked contrast to the usual observation that the aggregated species dissociate upon increasing temperature.^55–57^ Such a growth in the MMLCT absorption bands at *ca*. 620 nm is also observed in the variable-temperature UV–vis absorption spectra of **1** in acetonitrile–methanol (4:1 v/v), acetonitrile–isopropanol (4:1 v/v), and acetonitrile–*n*-butanol mixtures (4:1 v/v) upon increasing temperature (Figure S10a–c). To investigate the role of alcohol in the self-assembly process upon increasing temperature, variable-temperature UV–vis absorption studies of **1** in different acetonitrile–ethanol compositions have also been performed. Similar to the spectral change of **1** in acetonitrile–ethanol (4:1 v/v), an increase in the MMLCT absorption bands at *ca*. 620 nm is observed upon increasing the acetonitrile–ethanol content to 3:2 (v/v) (Figure S11a). However, further increasing the ethanol content results in a less drastic increase in the MMLCT absorption bands (Figure S11b–d). It is interesting to observe that the aggregation of the complex molecules upon heating significantly depends on the nature and proportion of alcohol in the solvent mixture.

**Figure 1:**
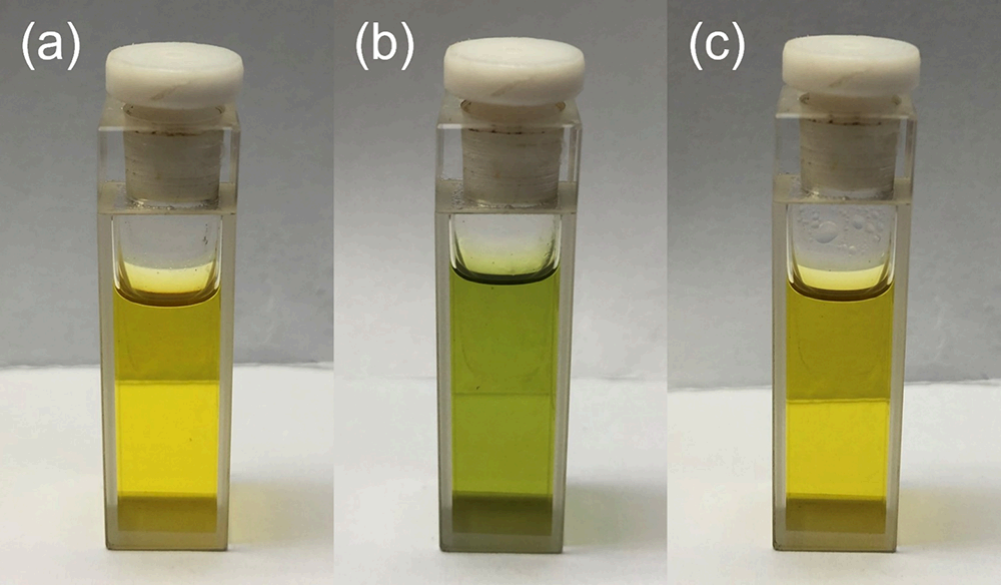
Solution of **1** in an acetonitrile–methanol mixture (4:1 v/v) (a) at room temperature, (b) upon heating, and (c) upon return to room temperature after heating.

**Figure 2:**
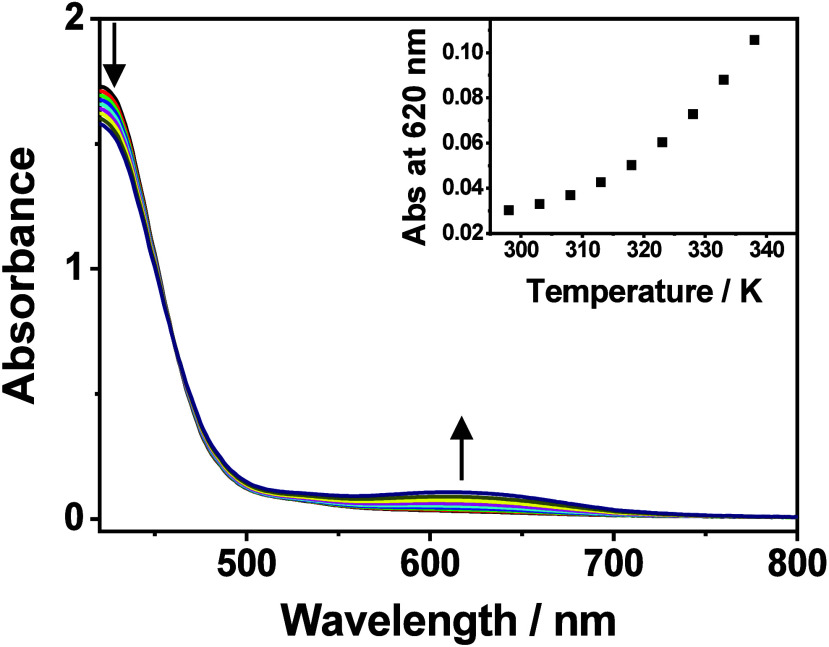
UV–Vis absorption spectra of **1** in acetonitrile–ethanol (4:1 v/v) ([Pt] = 811 μM) upon increasing temperature from 298 to 338 K. (Inset) A plot of absorbance at 620 nm against temperature.

Analogous to **1**, variable-temperature UV–vis absorption spectra of **2** and **3**, which consist of a hydrogen bond acceptor atom (i.e., N or O on both the alkynyl and pincer ligands), also show an increase in the MMLCT absorption band at about 620 nm upon increasing temperature in an acetonitrile–ethanol mixture (4:1 v/v) (Figures S12 and S13). Control complexes **4**–**6**, which lack an effective hydrogen bond acceptor atom on either the alkynyl or pincer ligand, demonstrate substantially different behaviors (Figures S14–S16). Despite the presence of potential hydrogen bond acceptor atoms (F) on the alkynyl ligand of **5**, the covalently bound F atoms, especially in −CF_3_ groups, are found to hardly act as hydrogen bond acceptors. Hence, the −CF_3_ group in **5** is not regarded as a hydrogen bonding moiety^58,59^ but rather mimics the electron deficiency of pyridine and pyrazine moieties. The necessity of the presence of hydrogen bond acceptor atoms on both the alkynyl and pincer ligands of the complexes as well as a hydrogen bond donor (alcohol) in the solvent mixture to induce those unusual spectroscopic changes suggests the potential contribution of the hydrogen bonding interaction to the change in aggregation properties of the complexes with increasing temperature.

In order to further investigate their assembly behavior, temperature-dependent ^1^H NMR experiments of **1** in the CD_3_CN–CD_3_OD mixture (4:1 v/v) have been performed (Figure 3). It is found that the aromatic proton signals of **1** are downfield shifted upon increasing the temperature from 298 to 318 K. The downfield shift of the aromatic proton signals is due to weakening of the shielding effect, which is caused by the adjacent aromatic units. This is indicative of the disruption of assemblies at this temperature. However, further increasing the temperature to 338 K causes an upfield shift and broadening of the aromatic proton signals. The upfield shift of the aromatic signals is due to the shielding effect, while the broadening of the signals can be attributed to the spin–spin relaxation effect resulting from the sample inhomogeneity and dynamic exchanges in the presence of aggregates. This suggests the unusual formation of another aggregate species upon increasing temperature, possibly attributed to a larger extent of Pt···Pt interactions as revealed by a more red-shifted low-energy absorption band at ca. 620 nm for **1** in acetonitrile–alcohol mixtures when compared to that at ca. 580 nm in pure acetonitrile at 298 K. This is in sharp contrast to the temperature-dependent ^1^H NMR experiments of structurally related control complex **6** in the CD_3_CN–CD_3_OD mixture (4:1 v/v). The aromatic proton signals of complex **6** are found to be downfield shifted and remained well-resolved upon increasing the temperature from 298 to 338 K (Figure S17), which is a typical observation for deaggregation in similar platinum­(II) systems that lack the hydrogen bond acceptor in the structure of the complexes.^22,60^ Moreover, the ^1^H NMR spectrum of **1** also shows downfield-shifted and well-resolved aromatic proton signals upon increasing the temperature in pure acetonitrile (Figures S18), suggesting the disruption of the possible self-assembly of platinum­(II) complexes under such conditions. This confirms the necessity of the presence of a hydrogen bond donor in the solvent mixture for the aggregation to occur with increasing temperature.

**Figure 3:**
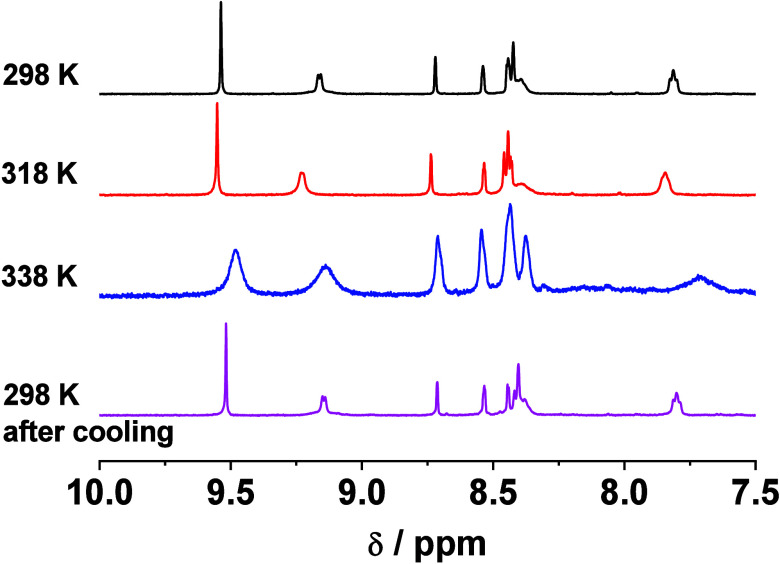
Partial ^1^H NMR spectra of **1** in CD_3_CN–CD_3_OD (4:1 v/v) in the aromatic region ([Pt] = 3770 μM) upon increasing the temperature from 298 to 338 K.

In order to verify the participation of hydrogen bonding in the aggregation of the system, acids and bases were added to the acetonitrile−ethanol mixture (4:1 v/v) of **1**. While the addition of hydrochloric acid (Figure S19) and methanesulfonic acid (Figure S20) leads to negligible changes in the lowest-energy absorption band, it is interesting to note that a similar phenomenon as that with the increasing temperature of **1** in an acetonitrile–ethanol mixture (4:1 v/v) (i.e., an increase in the absorption band at ca. 620 nm) is observed upon the addition of a base such as sodium hydroxide (NaOH) (Figure S21) or 1,8-diazabicyclo(5.4.0)­undec-7-ene (DBU) (Figure 4). Upon addition of acids, the nitrogen atoms on the complex are protonated as indicated by a slight blue shift of the MLCT/LLCT absorption band. This leads to the formation of a more positively charged and more electron-deficient species, which interacts more strongly with ethanol than that between acid and ethanol. In sharp contrast, the addition of base causes the breaking of hydrogen bonds between the complex and ethanol since the base outcompetes the complex molecules. This suggests a possible disruption of hydrogen bonding by the addition of a base or an increase in temperature to give similar color and spectroscopic changes. Further investigation through solution-state FT-IR spectroscopic studies supports the disruption of hydrogen bonding. The FT-IR spectra of both the mixed solvent alone containing an acetonitrile–ethanol mixture (4:1 v/v) with and without DBU (Figure S22) show strong distinct bands attributed to the O–H stretching mode of the ethanol molecules hydrogen bonded to acetonitrile (3530 cm^–1^).^61^ This indicates that the hydrogen bonds between the ethanol and acetonitrile molecules remain stable upon the addition of DBU. On the other hand, the background-subtracted FT-IR spectrum of **1** in the acetonitrile–ethanol mixture (4:1 v/v) without DBU (Figure S23) shows a strong distinct vibrational stretch at 3430 cm^–1^. This is attributed to the O–H bond stretching of a fraction of ethanol molecules hydrogen bonded to the complex molecules. Interestingly, this vibrational band is not observed upon addition of DBU to the acetonitrile–ethanol mixture (4:1 v/v) of **1**, indicating the disruption of hydrogen bonding between the ethanol and complex molecules.

**Figure 4:**
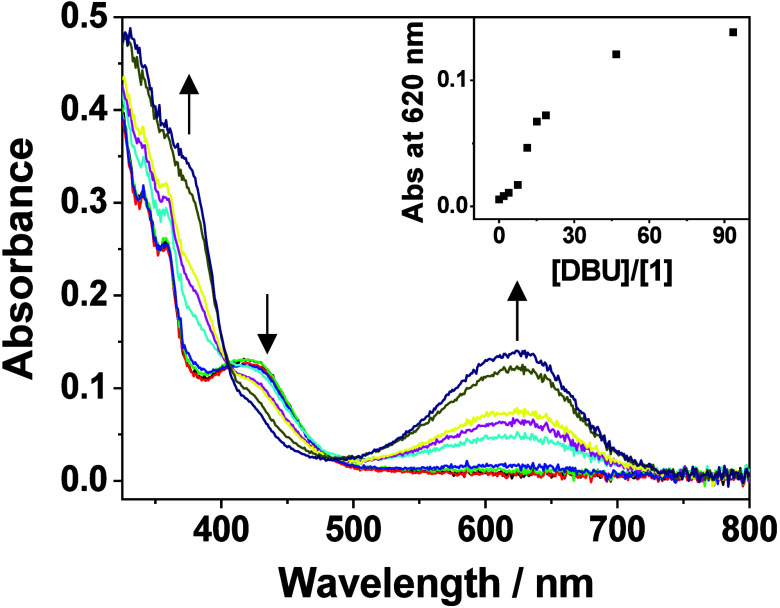
UV–Vis absorption spectra of **1** in an acetonitrile–ethanol mixture (4:1 v/v) ([Pt] = 18 μM) upon addition of DBU. (Inset) A plot of absorbance at 620 nm against the concentration ratio of DBU to **1**.

2D ^1^H–^1^H NOESY NMR experiments for **2** have been performed to elucidate the stacking arrangement between the complex cations at different temperatures in the CD_3_CN–CD_3_OD mixture (4:1 v/v). The ^1^H–^1^H NOESY NMR spectrum of **2** reveals that the complex cations are stacked in a slightly displaced head-to-tail arrangement with the platinum­(II) centers lying rather far apart from each other, as shown by the NOE signals between H_a_ and H_d_, H_e_ and H_c_, and H_a_ and H_e_ at 298 K (Figure S24). This packing mode of the complex cations resembles the crystal structure of the yellow form of **PtCl**, which involves long Pt···Pt distances, suggesting the small extent of Pt···Pt interactions among the aggregates in the CD_3_CN–CD_3_OD mixture at room temperature. Upon raising the temperature to 333 K, the complex cations are found to remain in a head-to-tail stacking fashion similar to that found in the X-ray crystal structures of the red form of **PtCl** and **2**, showing that the platinum­(II) centers of the complex dimers are close in proximity, as depicted by the appearance of NOE signals between H_a_ and H_f_ and between H_b_ and H_f_ (Figures S25). It is noteworthy that the NOE signals between H_a_ and H_d_, between H_e_ and H_c_, and between H_a_ and H_e_ have remained at 333 K, indicating a possible incomplete conversion between the aggregated species at this temperature. These results, together with the growth of a low-energy absorption band at ca. 620 nm at high temperature, indicate the presence of Pt···Pt interactions in the aggregates at 333 K.

To elucidate the origin of the unusual aggregation behavior at elevated temperature, atomistic MD simulations^62–65^ have been performed for complex **2** and its control complex **Ref** (Figure S26), which lacks hydrogen bond acceptors in both the pincer and alkynyl ligands. These simulations (Figure 5a) were carried out in an acetonitrile−ethanol mixture (4:1 v/v). Using the revised Lennard-Jones (LJ) force field parameters for platinum (σ­(Pt) = 0.33298 nm and *ε*(Pt) = 5.267 kJ mol^–1^), the intensity of the first *g*_PtPt_(*r*) peak of **Ref** at 298 K is slightly higher than (Figure S27d) or comparable to (Figure S31d) that at 350 K, suggesting less extensive Pt···Pt interactions at elevated temperatures. This agrees with the experimental findings in which the metal–metal-to-ligand charge transfer (MMLCT) absorption band of **Ref** remains nearly unchanged upon heating (Figure S29).

In sharp contrast to **Ref** (Figure S27d), complex **2** displays a significantly higher intensity in the first *g*_PtPt_(*r*) peak at 350 K compared to that at 298 K (Figure 5b), in line with the experimentally observed unusual aggregation behavior of **2** at elevated temperature. Due to the presence of hydrogen bond acceptors in **2**, the roles of the hydrogen bond in the self-assembly process of **2** are further investigated by statistical analysis (Figure 6). By utilizing the hydrogen bond acceptors (two nitrogen atoms) in **2** as the reference, RDF *g*_NH_(*r*) indicates the relative proton density (of the hydroxy group in ethanol) with respect to the average proton density within the simulation box. By plotting RDF *g*_NH_(*r*) as a function of the distance (*r*) from the reference, the most prominent peak appears at approximately *r* = 0.19 nm (Figure 6a), corresponding to the first solvation shell of ethanol. The higher *g*(*r* = 0.19 nm) value at 298 K compared to that at 350 K indicates that complex **2** is surrounded by more ethanol molecules at 298 K. Thus, there is a higher probability for the formation of N···H–O hydrogen bonds between **2** and ethanol molecules, thereby impeding the self-assembly of **2**. Moreover, it is found that the hydrogen bonds around **2** are stronger at 298 K than those at 350 K, as evidenced by the longer computed lifetime of hydrogen bonds (τ_HB_) surrounding **2** at 298 K (τ_HB_ = 7.24 ps) than at 350 K (τ_HB_ = 4.18 ps). The increased probability for N···H–O hydrogen bonds to give a smaller O···N distance (Figure 6b) and a more linear N···O–H angle (Figure 6c) at 298 K aligns with the increased stability of hydrogen bonds at lower temperature. Consistent results could also be derived from the replicated simulations with different initial configurations (Figure S32).

**Figure 5:**
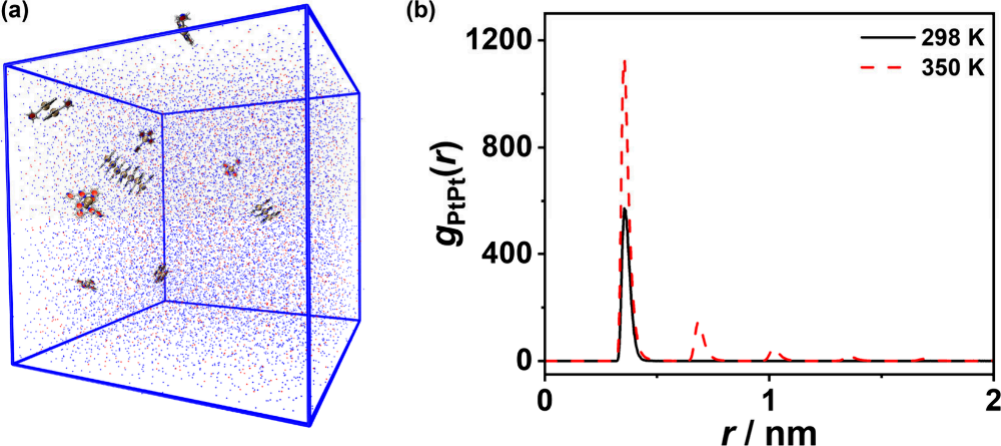
MD simulations for **2** in an acetonitrile–ethanol mixture (4:1 v/v). (a) The example simulation box demonstrating the configuration of **2** in an acetonitrile–ethanol mixture after a 200 ns simulation at 350 K. Blue and red dots represent acetonitrile and ethanol molecules, respectively. (b) *g*_PtPt_(*r*) curves at 298 and 350 K prepared using the trajectory in the range of 150–200 ns.

**Figure 6:**
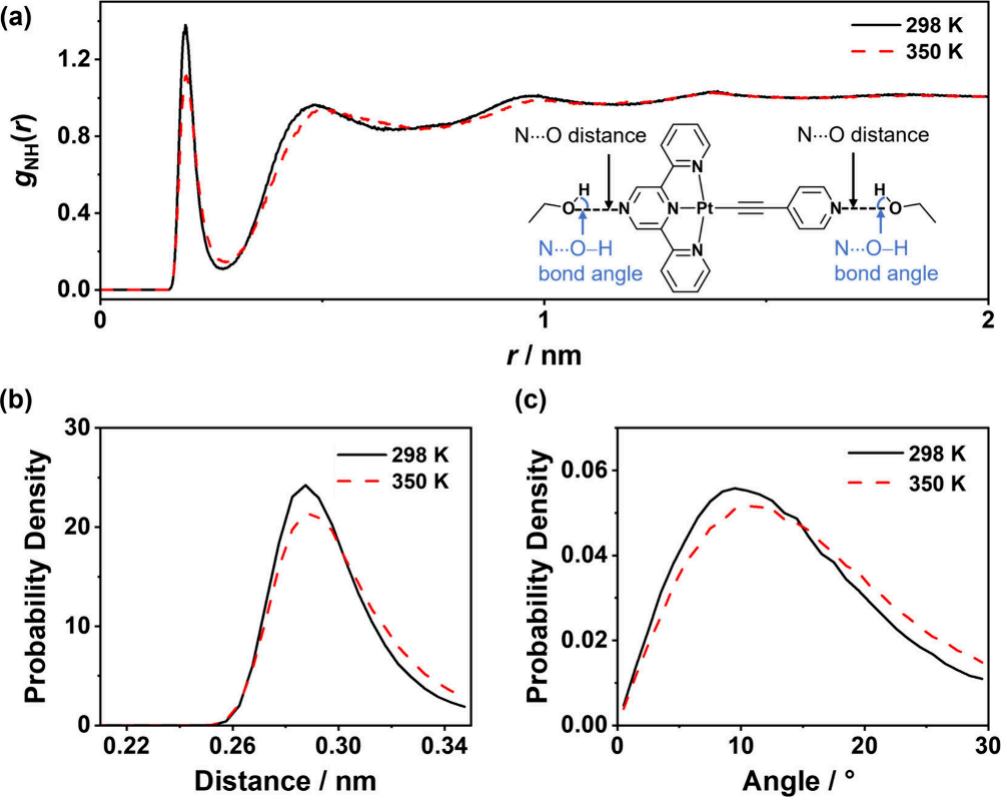
(a) Radial distribution function *g*_NH_(*r*) and distributions of the (b) N···O distance and (c) N···O–H angle for hydrogen bonds between **2** and ethanol.

In order to identify the morphology of the aggregated species, transmission electron microscopy (TEM) studies have been carried out. At 298 K, **1**–**3** are found to aggregate into rod-like structures with lengths of a few micrometers as indicated by the TEM images prepared from the acetonitrile–ethanol mixture (4:1 v/v) without heat treatment (Figures 7a, 8a, and S33a). The selected area electron diffraction (SAED) patterns of **1** and **2** display a pair of arc-shaped spots with a *d*-spacing of 0.34 nm (Figures 7b and S34b). This further supports the possible involvement of Pt···Pt and/or π–π stacking interactions along the long axis of the rod-like aggregates. At this temperature, slightly displaced head-to-tail alignment of the complex molecules is favored so that the hydrogen bond acceptor atoms on both the pincer and alkynyl ligands of the complex molecules can interact readily with the ethanol molecules. This agrees with the presence of hydrogen bonds between ethanol and complex molecules revealed by solution-state FT-IR and computational studies at room temperature. Upon an increase in temperature, the hydrogen bond between the ethanol and complex molecules is temporarily disrupted. The rod-like aggregates coil up to form circular morphologies (Figures 7c, 8b, and S33b) that are packed in a more compact head-to-tail manner through significant and more extensive Pt···Pt and π–π stacking interactions which are signaled by the establishment of the new lower-energy MMLCT absorption band and broadening of the ^1^H NMR signals. This is also consistent with the SAED pattern of the circular morphologies, which displays diffraction signals at a *d*-spacing of 0.33 nm (Figure S35b), as opposed to the 0.34 nm found before heat treatment, suggesting slightly closer Pt···Pt and/or π–π distances between the complex molecules at elevated temperature. Upon cooling, the hydrogen interactions between the complex and ethanol molecules would be recovered to give the thermodynamically more stable aggregates (Figures 7d, 8c, and S33c). Meanwhile, energy-dispersive X-ray (EDX) mapping analysis confirms the presence of Pt, N, S, and F elements in the corresponding platinum­(II) complexes in their rod-like and circular-like nanostructures (Figures S36–S38).

**Figure 7:**
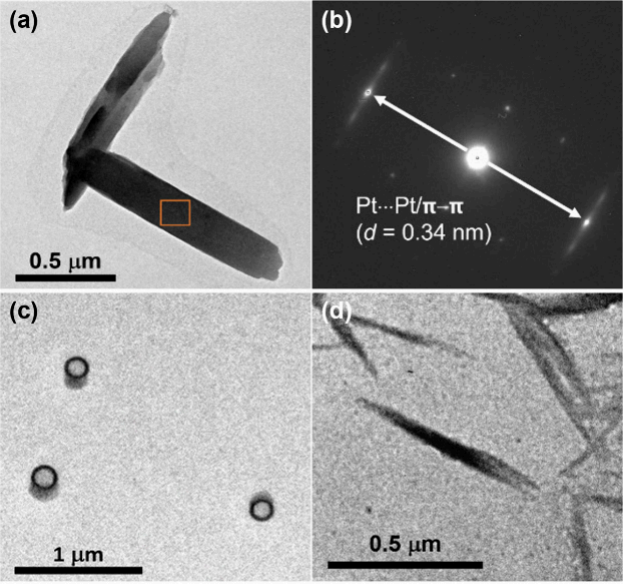
(a) TEM image and (b) SAED pattern of **1** in an acetonitrile–ethanol mixture (4:1 v/v) ([Pt] ≈ 10^–4^ M) without heat treatment. TEM images of **1** in an acetonitrile–ethanol mixture (4:1 v/v) ([Pt] ≈ 10^–4^ M) (c) with heat treatment and (d) after cooling.

**Figure 8:**
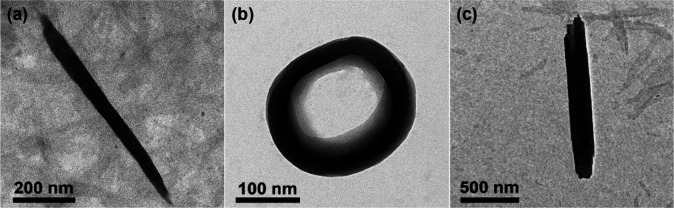
TEM images of **2** in an acetonitrile–ethanol mixture (4:1 v/v) ([Pt] ≈ 10^–4^ M) (a) without and (b) with heat treatment and (c) after cooling.

From the spectroscopic and computational results obtained, together with consideration of the possible hydrogen bonding ability of the complex molecules, a proposed mechanism for the high-temperature-induced aggregation is suggested. It is believed that acetonitrile is a relatively good solvent for **1** as suggested by the computational studies and structurally similar complexes in the previous reports.^16^ At room temperature, alcohols should solvate the complex cations via the hydrogen bond between the hydrogen bond donor (hydroxy group in the solvent) and the hydrogen bond acceptor atoms in the complex cations. Upon increasing temperature, the hydrogen bond between the alcohol and the complex cations is disrupted as supported by the solution-state FT-IR spectroscopy and computational studies, leading to the reduced solvation of the complex molecules at high temperatures, eventually resulting in stronger and more extensive self-assembly of the complexes brought about by Pt···Pt and π–π stacking interactions.^66^ This unique behavior shows the important roles of solvent and temperature in modulating the interplay of non-covalent interactions between the platinum­(II) complexes as well as between the solvent molecules and platinum­(II) complexes.

## Conclusions

A series of alkynylplatinum­(II) 2,6-(dipyrid-2-yl)­pyrazine complexes has been designed and synthesized. This class of complexes has been found to demonstrate drastic color change upon varying temperature due to thermoresponsive morphological transformation behavior in an acetonitrile−alcohol mixture via the interplay of Pt···Pt, π–π stacking, and hydrogen bonding interactions. At room temperature, the complexes have been found to self-assemble into rod-like structures via Pt···Pt and/or π–π interactions with the aid of the hydrogen bonding with alcohol molecules. Upon increasing temperature, the hydrogen bonds between the complexes and alcohol molecules are disrupted, leading to the formation of circular morphologies via the predominating Pt···Pt and π–π stacking interactions. The drastic color change of the complex solution upon the alteration of the temperature has provided insights into the development of thermochromic materials with platinum­(II) complexes via self-assembly. Moreover, with the demonstration of specific functional groups of the complex being necessary to facilitate the unique morphological change, this work has provided guiding principles for the rational design of stimuli-responsive assembly systems using platinum­(II) complexes.

## Supplementary Material



## Notes

General experimental details and procedures, computational details, X-ray crystallographic details, photophysical data, variable-temperature UV–vis absorption spectra, variable-temperature ^1^H NMR spectra, UV–vis absorption spectra upon addition of acid and base, variable-temperature FT-IR spectra, ^1^H–^1^H NOESY NMR spectra, computational results, and electron microscopy images (PDF)
