# Supplementary material for: Thermoresponsive Platinum(II) 2,6-Di(pyrid-2-yl)pyrazine Complexes with Unusual Aggregation Behavior upon Heating
Source: J Am Chem Soc. 2025 Jul 4;147(28):24941–9. doi: 10.1021/jacs.5c07413 (PMC12272684; doi:10.1021/jacs.5c07413)
Supplement: Supplementary file 1 [file ja5c07413_si_001.pdf]

Supporting Information

Thermoresponsive Platinum(II) 2,6-Di(pyrid-2-yl)pyrazine Complexes with Unusual Aggregation Behavior upon Heating

*Tracy Ho-Ying Chan, Ziyong Chen, Ming-Yi Leung, Michael Ho-Yeung Chan, Eric Ka-Ho Wong, Wai Kit Tang, Vivian Wing-Wah Yam**

Institute of Molecular Functional Materials and Department of Chemistry, The University of Hong Kong, Pokfulam Road, Hong Kong, P. R. China

E-mail: wwyam@hku.hk

Table of Contents

Physical Measurements and Instrumentation....3

Experimental Section....5

Computational Studies....8

X-ray Crystal Structures....12

Photophysical Studies....26

Variable-Temperature UV−vis Absorption Studies....27

Variable-Temperature ^{1}H NMR Spectra....35

UV−Vis Absorption Studies upon Addition of Acid and Base....36

Variable-Temperature FT-IR Spectra....38

^{1}H−^{1}H NOESY NMR spectra....39

Computational Results....40

Electron Microscopy....47

References....50

Physical Measurements and Instrumentation

^{1}H NMR and ^{1}H–^{1}H NOESY NMR spectra were recorded on a Bruker AVANCE 400 (400 MHz), a Bruker Ascend 500 (500 MHz), or a Bruker AVANCE 600 (600 MHz) Fourier-transform NMR spectrometer with chemical shifts relative to tetramethylsilane (Me_4Si). Positive-ion electrospray ionization (ESI) mass spectra were recorded on a Bruker maXis II high-resolution ESI-QTOF mass spectrometer. Elemental analyses were performed on a Flash EA 1112 elemental analyzer by the Institute of Chemistry at the Chinese Academy of Sciences in Beijing. The UV–visible absorption spectra were recorded on a Varian Cary 50 UV–vis spectrophotometer with the monitoring of the temperature using the Varian Cary single-cell Peltier thermostat. Concentration-dependent UV-vis absorption studies were carried out in quartz cuvettes with path lengths of 1 mm, 2 mm and 1 cm such that the absorbance would fall within the range of 0.1−1, following the validity of the Beer's Law. The apparent absorbance values were obtained by correcting to 1-cm path length equivalence. Single crystal X-ray diffraction (SCXRD) data of the crystals were collected using synchrotron radiation on beamline BL17B1 at the National Facility for Protein Science (NFPS) of the Shanghai Synchrotron Radiation Facility (SSRF), Shanghai. The reduction and integration of the diffraction data were performed by the HKL3000 software. The structures were solved by direct methods and refined employing full-matrix least-squares on F2 by using SHELXL program[1] through the OLEX2 interface.[2] Non-H atoms were located according to the direct methods. All non-H atoms of the complexes were refined with anisotropic thermal parameters. The hydrogen atoms were included in idealized positions and refined with fixed geometry with respect to their carrier atoms. The quality of the crystal data, disorders of solvent molecules and some counter-anions and large voids of the structures are responsible for the remaining A and B level Alerts observed in the checkcif reports. The disordered solvent molecules were removed using SQUEEZE routine of PLATON.[3] The X-ray crystallographic data for red and yellow form of **PtCl**, **2** and **5** have been deposited at the Cambridge Crystallographic Data Centre (CCDC), under the deposition number CCDC 2448300, CCDC 2448301, CCDC 2448302 and CCDC 2448303, respectively. Transmission electron microscopy (TEM) experiments were performed on a FEI Tecnai G2 20 S-TWIN TEM with an accelerating voltage of 200 kV or on a Philips CM100 TEM with an accelerating voltage of 100 kV. The selected area electron diffraction (SAED) experiments were performed on a FEI Tecnai G2 20 S-TWIN transmission electron microscope with an accelerating voltage of 200 kV. The

HAADF-STEM image and the elemental mapping results were obtained by thermo Scientific Talos F200X STEM. IR spectra were obtained in the solution state on a IRAffinity-1 Fourier transform infrared spectrophotometer (4000−500 cm^{-1}). To account for the background, the spectrum of the solvent mixture was subtracted from the sample spectrum. Hence, the vibrational stretch attributed to O–H bonds of the ethanol molecule hydrogen bonded to acetonitrile molecules (3530 cm^{-1}) is not observed in the FT-IR spectra of the samples.

Experimental Section

Materials and Reagents

Potassium tetrachloroplatinate(II) ($K_2[PtCl_4]$) (98 %) was purchased from Chem. Pur. 2,6-Dibromopyrazine (95 %) was purchased from Alfa Aesar Chemical Co. Ltd. 2,2′:6′,2″-Terpyridine (tpy) (98 %), 2-(tributylstannyl)pyridine (85 %), silver triflate (99 %), phenylacetylene (98 %), 2-iodopyrazine (97 %), 4-iodophenol (99 %), 1-bromododecane (97 %), 4-ethynylpyridine (98 %), 1.0 M tetra-*n*-butylammonium fluoride (TBAF) in THF and copper(I) iodide (98 %) were purchased from Sigma-Aldrich Co. Ltd. 4-(*t*-Fluoromethyl)phenylacetylene (97 %) was purchased from Apollo Scientific Ltd. Trimethylsilylacetylene (98 %) was purchased from GFS Chemical Co. Ltd. Triisopropylsilylacetylene (98 %) and trimethylamine were purchased from Fluorochem Ltd. All other solvents and commercially available reagents were of analytical grade and were used as received. Dichlorobis(triphenylphosphine)palladium(II),[4] tetrakis(triphenylphosphine)-palladium(0),[5] 2,6-di(pyrid-2-yl)pyrazine,[6] 2-ethynylpyrazine,[7] 1-(dodecyloxy)-4-ethynylbenzene,[8] $[Pt(DMSO)_2Cl_2]$[9], [Pt(tpy)Cl]OTf[10] and $[Pt(tpy)(C{\equiv}CC_6H_5)]OTf$[11] (**Ref**) were synthesized according to that reported in the literature. All reactions, unless specified otherwise, were carried out under an inert atmosphere of nitrogen using standard Schlenk techniques.

Synthesis

[Pt{2,6-(dipyrid-2-yl)pyrazine}Cl]OTf (**PtCl**). To a stirred solution of $[Pt(DMSO)_2Cl_2]$ (50 mg, 0.12 mmol) in acetone was added silver triflate (33 mg, 0.13 mmol) in acetone in a dropwise manner. The mixture was then stirred at room temperature for 30 minutes, and the resulting white suspension was filtered. The filtrate was added to 2,6-(dipyrid-2-yl)pyrazine (25 mg, 0.11 mmol) in acetone and the mixture was stirred at room temperature overnight. The reaction mixture was filtered and washed with diethyl ether. Subsequent recrystallization by slow diffusion of diethyl ether vapor into an acetonitrile solution of the product gave red crystals. Yield: 80 %. ^{1}H NMR (500 MHz, CD_3CN, 298 K): δ/ppm = 7.93–7.96 (m, 2H, pyridine), 8.39–8.47 (m, 4H, pyridine), 9.10 (d, 2H, J = 5.5 Hz, pyridine), 9.44 (s, 2H, pyrazine). Positive-ion HR-ESI-MS: m/z found ($[M-OTf]^+$ calcd. for $[C_{14}H_{10}ClN_4Pt]^+$) = 465.0217 (465.0229).

[Pt{2,6-(dipyrid-2-yl)pyrazine}C≡CC$_4$H$_3$N$_2$)]OTf (**1**). To a stirred solution of 2-ethynylpyrazine (41 mg, 0.39 mmol) in methanol was added potassium fluoride (45 mg, 0.78 mmol). The resultant solution was heated under reflux for 30 min. [Pt{2,6-(dipyrid-2-yl)pyrazine}Cl]OTf (200 mg, 0.32 mmol) was added to the reaction mixture, and the resulting orange solution was refluxed for 48 h. The solvent was evaporated under reduced pressure, and the product was extracted with acetonitrile. Recrystallization by diffusion of diethyl ether vapor into an acetonitrile solution of the product gave **1** as a brown solid. Yield: 60 %. ^{1}H NMR (500 MHz, CD$_3$CN, 298 K): δ/ppm = 9.52 (s, 2H, pyrazine), 9.14 (d, 2H, J = 5.4 Hz, pyridine), 8.71–8.72 (m, 1H, 2-ethynylpyrazine), 8.53–8.54 (m, 1H, 2-ethynylpyrazine), 8.43 (d, 1H, J = 2.5 Hz, 2-ethynylpyrazine), 8.38–8.42 (m, 4H, pyridine), 7.81–7.86 (m, 2H, pyridine). Positive-ion HR-ESI-MS: *m*/*z* found ([M−OTf]$^+$ calcd. for [C$_{20}$H$_{13}$N$_6$Pt]$^+$) = 532.0835 (532.0845). Elemental analysis calcd. (%) for C$_{21}$H$_{13}$F$_3$N$_6$O$_3$PtS: C 37.01, H 1.92, N 12.33; found: C 36.68, H 2.17, N 12.00.

[Pt{2,6-(dipyrid-2-yl)pyrazine}(C≡CC$_5$H$_4$N-4)]OTf (**2**). This was prepared according to a procedure similar to that described for **1**, except 4-ethynylpyridine (40 mg, 0.39 mmol) was used in place of 2-ethynylpyrazine. Yield: 62 %. ^{1}H NMR (500 MHz, CD$_3$CN, 298 K): δ/ppm = 9.53 (s, 2H, pyrazine), 9.21–9.25 (m, 2H, pyridine), 8.55 (d, 2H, J = 5.4 Hz, 4-ethynylpyridine), 8.41-8.43 (m, 4H, pyridine), 7.85–7.90 (m, 2H, pyridine), 7.42 (d, 2H, J = 5.4 Hz, 4-ethynylpyridine). Positive-ion HR-ESI-MS: *m*/*z* found ([M−OTf]$^+$ calcd. for [C$_{21}$H$_{14}$N$_5$Pt]$^+$) = 531.0852 (531.0893).

[Pt{2,6-(dipyrid-2-yl)pyrazine}(C≡CC$_6$H$_4$OC$_{12}$H$_{25}$-4)]OTf (**3**). This was prepared according to a procedure similar to that described for **1**, except 4-(dodecycloxy)phenylacetylene (112 mg, 0.39 mmol) was used in place of 2-ethynylpyrazine. Yield: 62 %. ^{1}H NMR (500 MHz, CD$_3$CN, 298 K): δ/ppm = 9.49 (s, 2H, pyrazine), 9.28–9.30 (m, 2H, pyridine), 8.38–8.44 (m, 4H, pyridine), 7.86–7.89 (m, 2H, pyridine), 7.45 (d, 2H, J = 8.6 Hz, 4-(dodecycloxy)phenylacetylene), 6.91 (d, 2H, J = 8.6 Hz, 4-(dodecycloxy)phenylacetylene), 4.01 (t, 2H, J = 6.0 Hz, –CH$_2$O), 3.40–3.45 (m, 2H, –CH$_2$–), 1.74–1.85 (m, 4H, –CH$_2$–), 1.43–1.50 (m, 2H, –CH$_2$–), 1.25–1.40 (m, 10H, –CH$_2$–), 1.10–1.13 (m, 2H, –CH$_2$–), 0.89 (t, 3H, J = 6.5 Hz, –CH$_3$). Positive-ion HR-ESI-MS: *m*/*z* found ([M−OTf]$^+$ calcd. for [C$_{34}$H$_{39}$N$_4$OPt]$^+$) = 714.2747 (714.2769).

[Pt{2,6-(dipyrid-2-yl)pyrazine}(C≡CC$_6$H$_5$)]OTf (**4**). This was prepared according to a procedure similar to that described for **1**, except phenylacetylene (40 mg, 0.39 mmol) was used in place of 2-ethynylpyrazine. Yield: 65 %. ^{1}H NMR (500 MHz, CD$_3$CN, 298 K): δ/ppm = 9.52 (s, 2H, pyrazine), 9.26–9.28 (m, 2H, pyridine), 8.40–8.43 (m, 4H, pyridine), 7.85–7.89 (m, 2H, pyridine), 7.53 (d, 2H, J = 7.7 Hz, phenylacetylene), 7.36–7.40 (m, 2H, phenylacetylene), 7.30 (t, 1H, J = 7.7 Hz, phenylacetylene). Positive-ion HR-ESI-MS: m/z found ([M–OTf]$^+$ calcd. for [C$_{22}$H$_{15}$N$_4$Pt]$^+$) = 530.0962 (530.0941).

[Pt{2,6-(dipyrid-2-yl)pyrazine}(C≡CC$_6$H$_4$CF$_3$-4)]OTf (**5**). This was prepared according to a procedure similar to that described for **1**, except 4-(trifluoromethyl)phenylacetylene (66 mg, 0.39 mmol) was used in place of 2-ethynylpyrazine. Yield: 62 %. ^{1}H NMR (500 MHz, CD$_3$CN, 298 K): δ/ppm = 9.52 (s, 2H, pyrazine), 9.27 (d, 2H, J = 4.8 Hz, pyridine), 8.39–8.45 (m, 4H, pyridine), 7.86–7.90 (m, 2H, pyridine), 7.68–7.70 (m, 4H, 4-(trifluoromethyl)phenylacetylene). Positive-ion HR-ESI-MS: m/z found ([M–OTf]$^+$ calcd. for [C$_{23}$H$_{14}$F$_3$N$_4$Pt]$^+$) = 598.0799 (598.0815). Elemental analysis calcd. (%) for C$_{24}$H$_{18}$F$_6$N$_4$O$_3$PtS: C 38.56, H 1.89, N 7.50; found: C 38.24, H 1.93, N 7.00.

[Pt{tpy}(C≡CC$_4$H$_3$N$_2$)]OTf (**6**). This was prepared according to a procedure similar to that described for **1**, except [Pt(tpy)Cl]OTf (200 mg, 0.32 mmol) was used in place of [Pt{2,6-(dipyrid-2-yl)pyrazine}Cl]OTf. Yield: 69 %. ^{1}H NMR (500 MHz, CD$_3$CN, 298 K): δ/ppm = 9.03 (d, 2H, J = 5.5 Hz, tpy), 8.68 (s, 1H, 2-ethynylpyrazine), 8.54–8.55 (m, 1H, 2-ethynylpyrazine), 8.42–8.44 (m, 1H, 2-ethynylpyrazine), 8.39 (t, 1H, J = 7.7 Hz, tpy), 8.29–8.34 (m, 2H, tpy), 8.22–8.27 (m, 4H, tpy), 7.71–7.74 (m, 2H, tpy). Positive-ion HR-ESI-MS: m/z found ([M–OTf]$^+$ calcd. for [C$_{21}$H$_{14}$N$_5$Pt]$^+$) = 531.0876 (531.0893).

Computational Studies

Validation of Force Field Parameters for Solvent Molecules

Properties of pure acetonitrile and ethanol solvent molecules have been simulated with atomistic molecular dynamics (MD) using the generalized AMBER force field (GAFF)[12] before modelling the self-assembly process. At room temperature, the experimental values (Table S14) are reproduced by MD simulations with acceptable error.

Optimization of Lennard-Jones Force Field Parameters for MD simulation of 2

MD simulations were first performed for **Ref** in acetonitrile–ethanol mixture (4:1, v/v) (Figure S26a), which is a control complex of **2** (Figure S26b) without hydrogen bond acceptors in both the pincer and alkynyl ligands. The pairwise radial distribution function (RDF) $g_{PtPt}(r)$, describing the distribution of Pt···Pt distances, was extracted from the post-simulation trajectory (Figures 5 and S27). An example radial distribution function (RDF), $g_{PtPt}(r)$, is shown in Figure S27c. The dominant peak at $r \sim 0.358$ nm signifies the existence of a dimeric aggregate (order $n = 2$) as well as dimeric structures within a higher-order aggregate ($n > 2$, Figure S28a). Similarly, the secondary peak at $r \sim 0.684$ nm corresponds to the Pt···Pt distance in a trimeric aggregate ($n = 3$) and trimeric structures in the higher-order aggregate ($n > 3$, Figure S28b). The intensity of the $g_{PtPt}(r)$ peak is proportional to the extent of Pt···Pt interactions. The intensity of the first peak was monitored over the simulation time t (Figures S27a and S27b). It is found that the intensity of the first peak becomes stable when $t > 150$ ns; thus, RDF curves in Figures S27c and S27d were prepared using the MD trajectory from 150–200 ns. With the previously reported LJ parameters (σ(Pt) = 0.33298 nm and ε(Pt) = 10.534 kJ mol^{-1}),[13] the first $g_{PtPt}(r)$ peak at 350 K is higher than that at 298 K (Figure S27c), indicating more extensive Pt···Pt interactions at 350 K. However, this contradicts experimental findings in which the metal–metal-to-ligand charge transfer (MMLCT) absorption band of **Ref** remains nearly unchanged, i.e. **Ref** does not exhibit a higher degree of aggregation, upon heating (Figure S29). Interestingly, upon reducing the strength of Pt···Pt interactions (ε(Pt) = 5.267 kJ mol^{-1}) in the MD simulations, the intensity of the first $g_{PtPt}(r)$ peak at 298 K becomes comparable to (Figure S31d) or slightly higher (Figure S27d) than that at 350 K. This aligns with the experimental result. Based on these results, it is proposed that ε(Pt) = 10.534 kJ mol^{-1} value might over-estimate the extent of Pt···Pt interactions between **Ref** molecules. At elevated temperatures, complexes move more rapidly and collide more vigorously, possibly leading to

an artificially increased likelihood of aggregate formation due to the over-estimated Pt···Pt interactions. It is anticipated that the Pt···Pt distances should be more dynamic at higher temperatures, while the over-estimation of Pt···Pt interactions might prevent temporary disruption of Pt···Pt interactions (Figure S30). To rectify this, the original ε(Pt) value is scaled by a factor of 0.5, leading to the increased first $g_{PtPt}(r)$ peak at 298 K compared to that at 350 K (Figure S27d). Further examination of Pt···Pt distances during the simulation at 350 K demonstrated that temporary disruption of Pt···Pt interactions could occur with the adjusted ε(Pt) parameter (Figure S30). In replicated simulations with a different initial configuration (Figure S31), it is also noticed that the adoption of ε(Pt) = 5.267 kJ mol^{-1} leads to a similar intensity of the first $g_{PtPt}(r)$ peak at both 298 K and 350 K (Figure S31d), which mitigates the over-estimation of Pt···Pt interactions when using ε(Pt) = 10.534 kJ mol^{-1} (Figure S31c). As such, the revised LJ parameters (σ(Pt) = 0.33298 nm and ε(Pt) = 5.267 kJ mol^{-1}) have been implemented in the MD simulations of **2**.

Computational Details

Quantum Chemical Calculations

All density functional theory (DFT) calculations were performed using Gaussian 16 (revision A.03) package.[14] The optimized ground state (S_0) geometries of solvent molecules (ethanol and acetonitrile), complex **2** and the control complex, **Ref**, were computed at PBE0 level in conjunction with the D3 version of Grimme's dispersion with Becke-Johnson damping (PBE0-D3BJ).[15] The Stuttgart effective core potentials (SDD) and the associated basis set were used to describe platinum,[16] whereas the 6-31G(d,p) basis set was used for all other atoms. The bulk solvent effect in the acetonitrile environment was simulated using the polarizable continuum model (PCM).[17] Vibrational frequencies were computed to verify that each stationary point was a minimum on the potential energy surface (NIMAG = 0). Restrained electrostatic potential (RESP) atomic charges[18] were also computed using Multiwfn (version 3.8dev) package[19] for subsequent atomistic MD simulations. Cartesian coordinates of the optimized S_0 state structures of complex **2** and **Ref** are provided in Tables S15 and S16, respectively.

Molecular Force Field

GAFF parameters were used to describe the bonded and non-bonded interactions between solvent molecules (ethanol and acetonitrile) and chloride counterions. For complexes **2** and **Ref**, the systems have been divided into rigid (pincer ligand and pyridinyl/phenyl in the

monodentate ligand) and flexible (Pt–C≡C–C linker) parts. The bonded interactions in the rigid part were obtained using the modified Seminario method,[20] which derives accurate harmonic bond, angle and dihedral parameters directly from the PBE0-D3BJ computed Hessian matrix. The bonded interactions in the flexible part were described by GAFF, which allows the rotation of the C≡C bond. GAFF was also applied to describe the non-bonded interactions for the non-metal part in complexes **2** and **Ref**. The LJ parameters (σ(Pt) = 0.33298 nm and ε(Pt) = 5.267 kJ mol^{-1} or 10.534 kJ mol^{-1}) for platinum were used to properly model the metal–metal interactions in the self-assembly process. RESP atomic charges computed at PBE0-D3BJ level were used. All topology files were generated by Sobtop (version 1.0, dev 3.1) package.[21]

MD Simulations of Pure Ethanol and Acetonitrile Solvents

All MD simulations were performed using GROMACS (version 2021.3) package.[22] The initial configuration were generated by randomly placing 4000 ethanol/acetonitrile molecules in a cubic simulation box with an edge length of 8 nm. The energy minimization (EM) was performed (maximal force F_{max} < 100 kJ·mol^{-1}·nm^{-1} on each atom), followed by a 2 ns equilibrium simulation under the normal pressure and temperature (298 K, 1 atm) ensemble using V-rescale thermostat and Berendsen barostat (isothermal compressibility $\kappa = 1.2 \times 10^{-4}$ bar^{-1}) with the coupling time constant of 0.2 and 2 ps, respectively. The temperature T was increased from 0 to 298 K during the initial 1 ns of the simulation, and then T was maintained at 298 K. The long-range electrostatic interactions beyond the cutoff at 10 Å were considered using the particle-mesh Ewald (PME) method, and the integration time step was 2 fs. Finally, a 10 ns production simulation was performed under the normal pressure and temperature (298 K, 1 atm) ensemble using V-rescale thermostat and Parrinello-Rahman barostat ($\kappa = 1.2 \times 10^{-4}$ bar^{-1}) with the coupling time constant of 0.2 and 2 ps, respectively. The integration time step was set to 1 fs in the production simulation.

MD Simulations of 2 and Ref in Acetonitrile–Ethanol Mixture

For complex **2** in acetonitrile–ethanol mixture (4:1, v/v), the initial configuration were generated by randomly placing 20 complex cations, 20 chloride counteranions, 13076 acetonitrile molecules and 2924 ethanol molecules in a cubic simulation box with an edge length of 13 nm. The EM was performed, followed by a 2 ns equilibrium simulation under the normal pressure and temperature (298 K or 350 K, 1 atm) ensemble using V-rescale thermostat and Berendsen barostat ($\kappa = 1.2 \times 10^{-4}$ bar^{-1}) with the coupling time constant of 0.2 and 2 ps, respectively. T was increased from 0 to 298 K (or 350 K) during the initial 1 ns of the simulation, and then T was maintained at 298 K (or 350 K). Finally, a 200 ns production simulation was performed under the normal pressure and temperature ensemble at 298 K (or 350 K) and 1 atm. Other simulation details were the same as the MD simulations of pure ethanol and acetonitrile solvents. Two replicated simulations with different initial configurations were performed for each temperature. MD simulations of **Ref** adopted similar settings as those of **2**. To make a fair comparison, simulations at 298 K/350 K share the same initial configuration for each replicated simulation. For hydrogen bonds between complex **2** and ethanol (Figure 6a), it is required that the distance between hydrogen bond acceptor and donor (N···O distance) is less than 0.35 nm, and the hydrogen bond cannot deviate too much from the linear configuration (N···O–H angle < 30°).

X-ray Crystal Structures

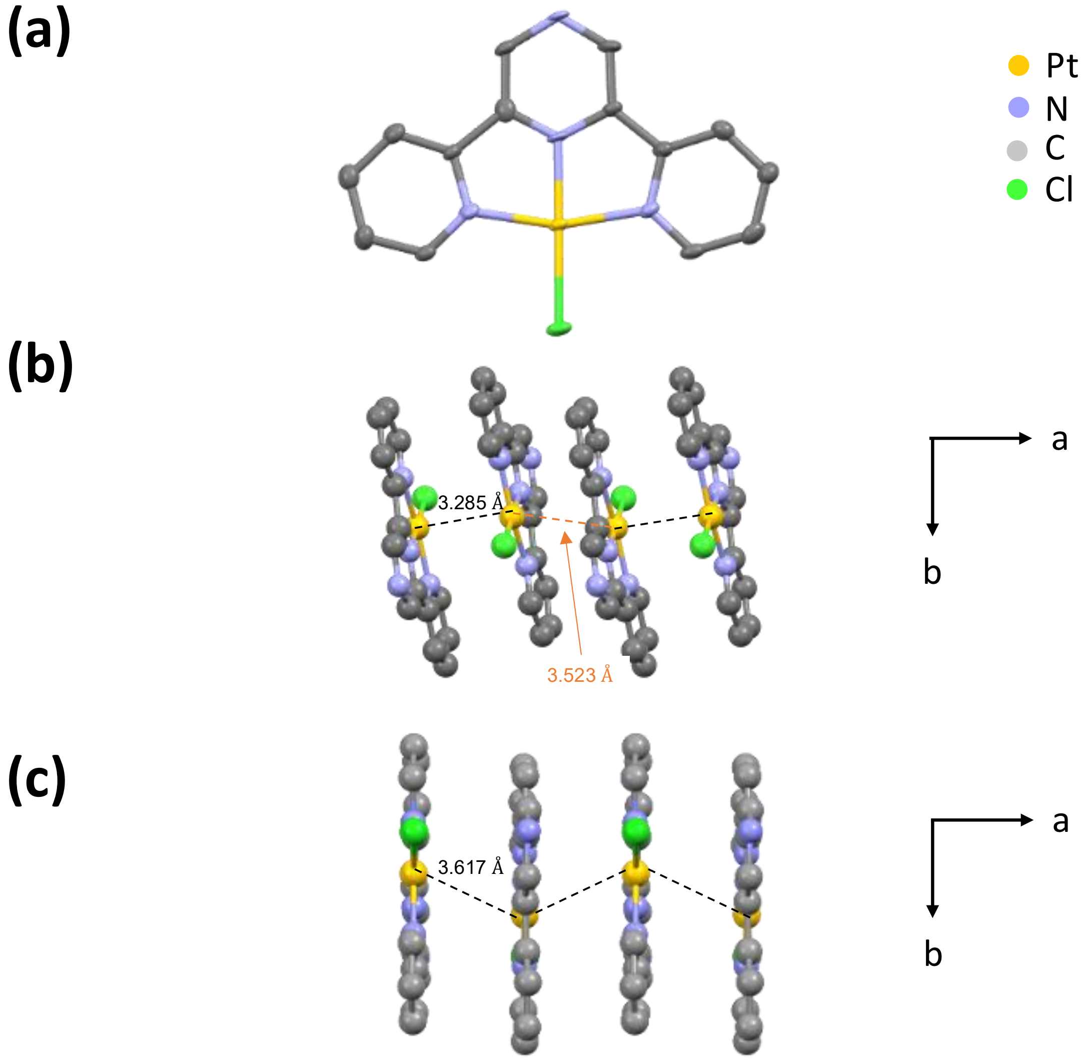

Figure S1. (a) Perspective view of **PtCl** with thermal ellipsoids at 50 % probability level. Crystal packing diagram of complex cation of **PtCl** in (b) red form and in (c) yellow form, both exhibiting zigzag arrangement. Hydrogen atoms and counter anions are omitted for clarity.

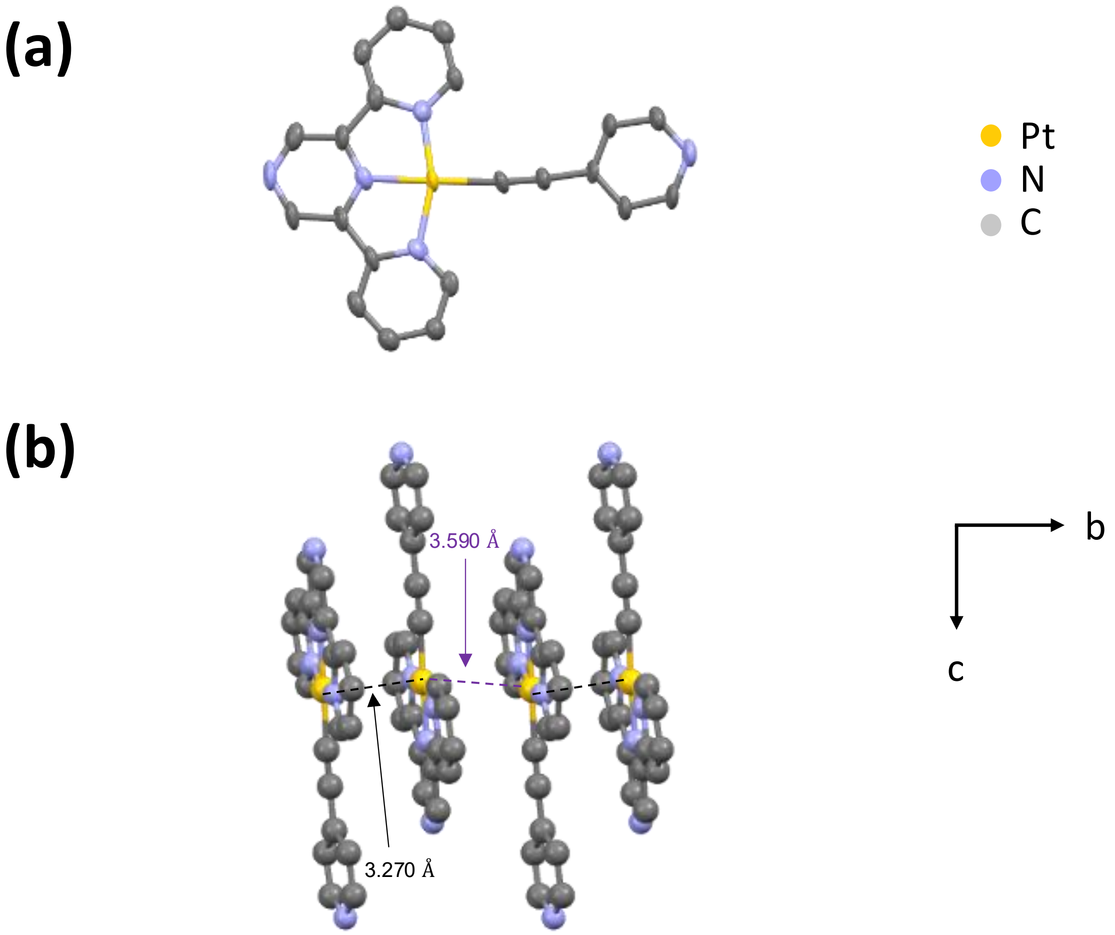

Figure S2. (a) Perspective view of complex cation of **2**. (b) Crystal packing of complex cation of **2** showing exhibiting zigzag arrangement. Hydrogen atoms and counter anions are omitted for clarity.

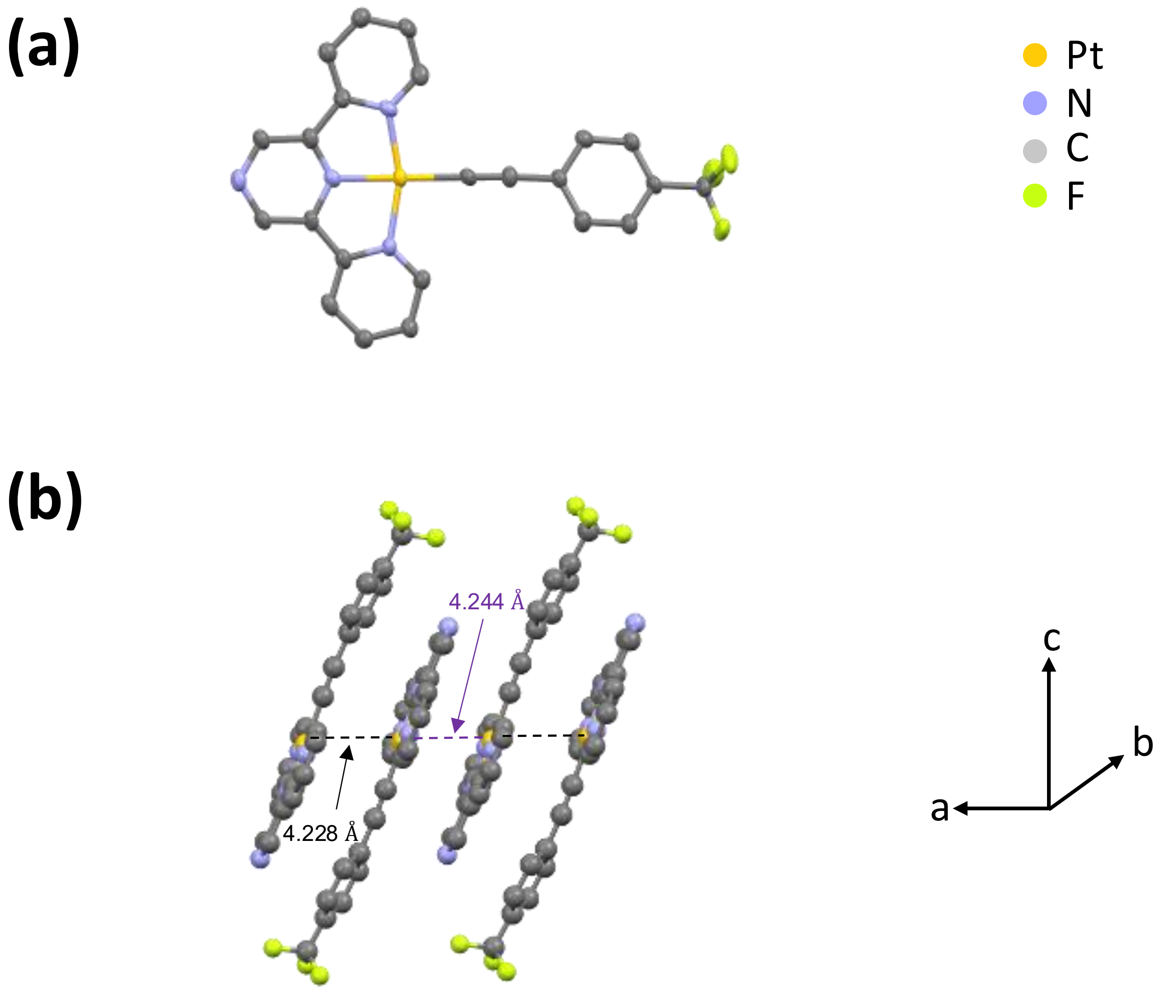

Figure S3. (a) Perspective view of complex cation of **5**. (b) Crystal packing of complex cation of **5** showing exhibiting zigzag arrangement. Hydrogen atoms and counter anions are omitted for clarity.

Table S1. Crystallographic and structural refinement data for red form of **PtCl**

Empirical formula	$C_{15}H_{10}ClF_3N_4O_3PtS$
Formula weight	613.87
Temperature/K	100.00
Crystal system	monoclinic
Space group	$P2_1/n$
a/Å	6.7587(4)
b/Å	14.5076(10)
c/Å	18.2714(12)

α/°	90
β/°	96.161(2)
γ/°	90
Volume/Å^3	1781.2(2)
Z	4
ρ_{calc} g/cm^3	2.289
μ/mm^{-1}	17.755
F(000)	1160.0
Crystal size/mm^3	0.04 mm × 0.01 mm × 0.01 mm
Radiation	Cu$K\alpha$ (λ = 1.54178 Å)
2Θ range for data collection/°	7.798 to 149.262
Index ranges	$-8 \leq h \leq 8$, $-18 \leq k \leq 14$, $-22 \leq l \leq 22$
Reflections collected	19961
Independent reflections	3633 [R_{int} = 0.0645, R_{sigma} = 0.0494]
Data/restraints/parameters	3633/0/253
Goodness-of-fit on F^2	1.237
Final R indexes [$I \geq 2\sigma(I)$]	R_1 = 0.0668, wR_2 = 0.1827
Final R indexes [all data]	R_1 = 0.0684, wR_2 = 0.1836

Table S2. Selected bond lengths (Å) for red form of **PtCl**

Pt1–Cl1	2.296(3)	N9–C10	1.35(2)
Pt1–N1	2.037(11)	N9–C8	1.34(2)
Pt1–N12	1.930(10)	C6–C5	1.36(2)
Pt1–N18	2.045(10)	C6–C7	1.481(18)
S19–O21	1.416(12)	C13–C11	1.501(18)
S19–O20	1.400(15)	C13–C14	1.354(19)
S19–O22	1.426(14)	F26–C23	1.26(3)
S19–C23	1.81(2)	F25–C23	1.30(2)
N1–C2	1.342(16)	C5–C4	1.40(2)
N1–C6	1.377(16)	C16–C17	1.396(19)
C2–C3	1.39(2)	C16–C15	1.40(2)

N12–C11	1.297(17)	F24–C23	1.37(3)
N12–C7	1.348(16)	C10–C11	1.401(17)
N18–C13	1.351(16)	C7–C8	1.386(17)
N18–C17	1.324(16)	C14–C15	1.39(2)
C3–C4	1.35(2)		

Table S3. Selected bond angles (°) for red form of **PtCl**

N1–Pt1–Cl1	99.2(3)	C5–C6–N1	120.8(12)
N1–Pt1–N18	161.9(4)	C5–C6–C7	125.3(12)
N12–Pt1–Cl1	178.6(3)	N18–C13–C11	112.9(11)
N12–Pt1–N1	81.4(4)	N18–C13–C14	123.3(12)
N12–Pt1–N18	80.5(4)	C14–C13–C11	123.8(12)
N18–Pt1–Cl1	98.9(3)	C6–C5–C4	119.5(14)
O21–S19–O22	115.2(9)	C17–C16–C15	118.2(13)
O21–S19–C23	103.0(10)	N18–C17–C16	121.0(12)
O20–S19–O21	116.2(9)	C3–C4–C5	118.3(15)
O20–S19–O22	115.0(10)	N9–C10–C11	121.4(13)
O20–S19–C23	104.0(11)	N12–C11–C13	114.8(11)
O22–S19–C23	100.4(12)	N12–C11–C10	117.7(12)
C2–N1–Pt1	126.5(9)	C10–C11–C13	127.5(13)
C2–N1–C6	120.5(12)	N12–C7–C6	114.0(11)
C6–N1–Pt1	113.0(8)	N12–C7–C8	117.5(13)
N1–C2–C3	118.9(13)	C8–C7–C6	128.5(13)
C11–N12–Pt1	118.4(9)	C13–C14–C15	117.4(13)
C11–N12–C7	123.8(11)	N9–C8–C7	121.5(14)
C7–N12–Pt1	117.7(8)	C14–C15–C16	120.0(13)
C13–N18–Pt1	113.4(8)	F26–C23–S19	112.6(16)
C17–N18–Pt1	126.5(8)	F26–C23–F25	112(2)
C17–N18–C13	120.0(11)	F26–C23–F24	106(2)
C4–C3–C2	121.9(14)	F25–C23–S19	113.5(17)

C8–N9–C10	118.0(11)	F25–C23–F24	104.2(19)
N1–C6–C7	113.9(12)	F24–C23–S19	107.9(16)

Table S4. Crystallographic and structural refinement data for yellow form of **PtCl**

Empirical formula	$C_{15}H_{10}ClF_3N_4O_3PtS$
Formula weight	613.87
Temperature/K	100.00
Crystal system	orthorhombic
Space group	$P2_12_12_1$
a/Å	6.7331(5)
b/Å	15.1987(13)
c/Å	16.8874(12)
α/°	90
β/°	90
γ/°	90
Volume/Å^3	1728.2(2)
Z	4
ρ_{calc} g/cm^3	2.359
μ/mm^{-1}	12.810
F(000)	1160.0
Crystal size/mm^3	0.04 mm × 0.01 mm × 0.01 mm
Radiation	Ga$K\alpha$ (λ = 1.34139 Å)
2Θ range for data collection/°	6.808 to 114.304
Index ranges	$-8 \leq h \leq 8, -18 \leq k \leq 18, -21 \leq l \leq 20$
Reflections collected	37074
Independent reflections	3547 [R_{int} = 0.0905, R_{sigma} = 0.0386]
Data/restraints/parameters	3547/156/256
Goodness-of-fit on F^2	1.251
Final R indexes [$I \geq 2\sigma(I)$]	R_1 = 0.1182, wR_2 = 0.2807
Final R indexes [all data]	R_1 = 0.1225, wR_2 = 0.2828

Table S5. Selected bond lengths (Å) for yellow form of **PtCl**

Pt1–Cl1	2.279(10)	N12–C11	1.39
Pt1–N18	2.11(2)	N12–C7	1.39
Pt1–N1	2.004(15)	C11–C10	1.39
Pt1–N12	1.87(2)	C10–N9	1.39
N18–C17	1.39	N9–C8	1.39
N18–C13	1.39	C8–C7	1.39
C17–C16	1.39	S1–O2	1.40(5)
C16–C15	1.39	S1–O1	1.39(7)
C15–C14	1.39	S1–O3	1.41(4)
C14–C13	1.39	S1–C19	1.78(7)
C13–C11	1.54(3)	S1–O1A	1.41(9)
N1–C6	1.39	S1–O3A	1.40(6)
N1–C2	1.39	F1–C19	1.25(8)
C6–C5	1.39	F3A–C19	1.12(10)
C6–C7	1.44(3)	F2A–C19	1.46(9)
C5–C4	1.39	F3–C19	1.71(10)
C4–C3	1.39	F2–C19	1.26(10)
C3–C2	1.39		

Table S6. Selected bond angles (°) for yellow form of **PtCl**

N18–Pt1–Cl1	97.7(8)	N12–C11–C10	120
N1–Pt1–Cl1	100.2(6)	C10–C11–C13	127(2)
N1–Pt1–N18	161.9(9)	N9–C10–C11	120
N12–Pt1–Cl1	179.3(11)	C10–N9–C8	120
N12–Pt1–N18	81.8(12)	N9–C8–C7	120
N12–Pt1–N1	80.2(10)	N12–C7–C6	113(2)
C17–N18–Pt1	127.5(15)	C8–C7–C6	127(2)
C17–N18–C13	120	C8–C7–N12	120
C13–N18–Pt1	112.4(15)	O2–S1–O3	103(3)
C16–C17–N18	120	O2–S1–C19	105(4)
C17–C16–C15	120	O2–S1–O1A	90(6)

C14–C15–C16	120	O1–S1–O2	124(4)
C15–C14–C13	120	O1–S1–O3	114(4)
N18–C13–C11	113(2)	O1–S1–C19	107(4)
C14–C13–N18	120	O3–S1–C19	100(3)
C14–C13–C11	127(2)	O1A–S1–C19	91(4)
C6–N1–Pt1	115.8(10)	O3A–S1–O2	137(4)
C6–N1–C2	120	O3A–S1–C19	110(4)
C2–N1–Pt1	124.2(10)	O3A–S1–O1A	112(6)
N1–C6–C7	111.3(17)	F1–C19–S1	117(6)
C5–C6–N1	120	F1–C19–F2A	95(6)
C5–C6–C7	128.7(17)	F1–C19–F3	95(5)
C6–C5–C4	120	F1–C19–F2	128(7)
C3–C4–C5	120	F3A–C19–S1	123(7)
C4–C3–C2	120	F3A–C19–F1	103(7)
C3–C2–N1	120	F3A–C19–F2A	104(7)
C11–N12–Pt1	120.4(16)	F2A–C19–S1	110(5)
C11–N12–C7	120	F3–C19–S1	98(4)
C7–N12–Pt1	119.5(16)	F2–C19–S1	114(6)
N12–C11–C13	113(2)	F2–C19–F3	85(6)

Table S7. Crystallographic and structural refinement data for complex **2**

Empirical formula	$C_{22}H_{14}F_3N_5O_3PtS \cdot CH_3CN$
Formula weight	721.58
Temperature/K	100.00
Crystal system	monoclinic
Space group	*C2/c*
a/Å	25.912(7)
b/Å	6.7420(14)
c/Å	28.144(6)
α/°	90
β/°	96.51(3)
γ/°	90

Volume/Å^3	4885(2)
Z	8
ρ_{calc} g/cm^3	1.962
μ/mm^{-1}	5.418
F(000)	2784.0
Crystal size/mm^3	0.03 mm × 0.01 mm × 0.01 mm
Radiation	synchrotron (λ = 0.68873 Å)
2Θ range for data collection/°	6.176 to 53.17
Index ranges	$-33 \le h \le 33$, $0 \le k \le 8$, $-36 \le l \le 36$
Reflections collected	8874
Independent reflections	5180 [R_{int} = 0.1511, R_{sigma} = 0.1179]
Data/restraints/parameters	5180/0/316
Goodness-of-fit on F^2	1.101
Final R indexes [$I \ge 2\sigma(I)$]	R_1 = 0.0884, wR_2 = 0.2620
Final R indexes [all data]	R_1 = 0.0945, wR_2 = 0.2718

Table S8. Selected bond lengths (Å) for complex **2**

Pt1–N18	2.049(8)	C13–C11	1.454(14)
Pt1–N1	2.046(7)	C13–C14	1.397(11)
Pt1–N12	1.969(14)	C7–C6	1.495(15)
Pt1–C19	1.993(15)	C7–C8	1.371(19)
N18–C17	1.337(12)	C22–C23	1.405(14)
N18–C13	1.370(12)	C14–C15	1.374(12)
N1–C2	1.334(10)	C16–C15	1.370(12)
N1–C6	1.402(11)	C25–C26	1.385(11)
N12–C7	1.339(17)	C6–C5	1.380(12)
N12–C11	1.358(16)	C5–C4	1.384(12)
N9–C10	1.337(16)	C3–C4	1.387(13)
N9–C8	1.34(2)	S1–O3	1.423(8)
N24–C23	1.352(13)	S1–O1	1.429(11)
N24–C25	1.345(10)	S1–O2	1.471(12)
C19–C20	1.199(19)	S1–C27	1.829(15)

C17–C16	1.399(11)	F1–C27	1.339(18)
C10–C11	1.407(13)	F3–C27	1.292(18)
C21–C20	1.438(13)	F2–C27	1.396(14)
C21–C22	1.397(14)	N28–C29	1.11(2)
C21–C26	1.402(12)	C29–C30	1.46(3)
C2–C3	1.387(12)		

Table S9. Selected bond angles (°) for complex **2**

N1–Pt1–N18	160.5(4)	N12–C11–C10	115.8(11)
N12–Pt1–N18	80.2(4)	N12–C11–C13	113.9(10)
N12–Pt1–N1	80.3(4)	C10–C11–C13	130.2(9)
N12–Pt1–C19	177.9(3)	C21–C22–C23	118.5(10)
C19–Pt1–N18	101.2(5)	C15–C14–C13	119.8(8)
C19–Pt1–N1	98.3(4)	C15–C16–C17	120.1(8)
C17–N18–Pt1	126.1(7)	N24–C23–C22	124.5(9)
C17–N18–C13	120.7(8)	N24–C25–C26	124.3(8)
C13–N18–Pt1	113.3(6)	N1–C6–C7	114.6(8)
C2–N1–Pt1	127.8(6)	C5–C6–N1	120.4(8)
C2–N1–C6	119.1(7)	C5–C6–C7	124.9(8)
C6–N1–Pt1	113.1(6)	C6–C5–C4	119.3(8)
C7–N12–Pt1	119.6(9)	C25–C26–C21	119.6(8)
C7–N12–C11	123.2(12)	C16–C15–C14	119.3(7)
C11–N12–Pt1	117.2(9)	N9–C8–C7	121.3(13)
C8–N9–C10	119.5(9)	C2–C3–C4	118.9(8)
C25–N24–C23	115.7(7)	C5–C4–C3	120.0(8)
C20–C19–Pt1	174.4(11)	O3–S1–O1	116.8(6)
N18–C17–C16	120.3(8)	O3–S1–O2	114.3(6)
N9–C10–C11	121.7(10)	O3–S1–C27	103.5(6)
C22–C21–C20	123.2(8)	O1–S1–O2	115.6(7)
C22–C21–C26	117.5(8)	O1–S1–C27	103.8(7)

C26–C21–C20	119.4(8)	O2–S1–C27	99.7(7)
N1–C2–C3	122.1(8)	F1–C27–S1	111.7(10)
N18–C13–C11	115.3(8)	F1–C27–F2	107.5(11)
N18–C13–C14	119.7(8)	F3–C27–S1	113.3(10)
C14–C13–C11	124.9(8)	F3–C27–F1	108.8(11)
N12–C7–C6	112.4(10)	F3–C27–F2	106.5(12)
N12–C7–C8	118.4(12)	F2–C27–S1	108.6(10)
C8–C7–C6	129.3(12)	N28–C29–C30	176.8(19)
C19–C20–C21	175.9(11)		

Table S10. Crystallographic and structural refinement data for complex **5**

Empirical formula	$C_{24}H_{14}F_6N_4O_3PtS$
Formula weight	747.54
Temperature/K	100.00
Crystal system	monoclinic
Space group	$P2_1/n$
a/Å	7.1700(14)
b/Å	18.105(4)
c/Å	18.053(4)
α/°	90
β/°	90.77(3)
γ/°	90
Volume/Å^3	2343.3(8)
Z	4
ρ_{calc} g/cm^3	2.119
μ/mm^{-1}	5.665
F(000)	1432.0
Crystal size/mm^3	0.04 mm × 0.02 mm × 0.01 mm
Radiation	synchrotron (λ = 0.68878 Å)
2Θ range for data collection/°	4.878 to 52.458
Index ranges	$-9 \le h \le 0$, $-22 \le k \le 22$, $-22 \le l \le 23$
Reflections collected	7381

Independent reflections	4421 [R_{int} = 0.1171, R_{sigma} = 0.0839]
Data/restraints/parameters	4421/0/353
Goodness-of-fit on F^2	1.084
Final R indexes [$I \geq 2\sigma(I)$]	R_1 = 0.0848, wR_2 = 0.2334
Final R indexes [all data]	R_1 = 0.0856, wR_2 = 0.2349

Table S11. Selected bond lengths (Å) for complex **5**

Pt1–N1	2.037(6)	N12–C11	1.372(9)
Pt1–N12	1.953(6)	C26–C25	1.365(10)
Pt1–N18	2.022(7)	C26–C21	1.422(9)
Pt1–C19	1.975(7)	C23–C22	1.381(10)
S1–O3	1.449(5)	N18–C13	1.369(9)
S1–O2	1.451(4)	N18–C17	1.352(10)
S1–O1	1.440(5)	C4–C3	1.380(10)
S1–C28	1.820(8)	C4–C5	1.399(11)
F2–C27	1.356(10)	C22–C21	1.411(9)
F3–C27	1.326(11)	N9–C10	1.336(9)
F1–C27	1.349(9)	N9–C8	1.350(9)
F5–C28	1.344(7)	C21–C20	1.442(9)
C16–C15	1.362(9)	C10–C11	1.402(10)
C16–C17	1.420(11)	C13–C11	1.456(10)
F6–C28	1.351(8)	C13–C14	1.387(10)
F4–C28	1.334(7)	C3–C2	1.397(9)
C24–C27	1.473(11)	C20–C19	1.200(10)
C24–C23	1.397(9)	C7–C6	1.486(10)
C24–C25	1.405(10)	C7–C8	1.420(9)
N1–C6	1.361(10)	C6–C5	1.384(9)
N1–C2	1.343(9)	C14–C15	1.399(10)
N12–C7	1.325(9)		

Table S12. Selected bond angles (°) for complex **5**

N12–Pt1–N1	80.4(2)	F4–C28–F5	107.3(5)
N12–Pt1–N18	81.0(2)	F4–C28–F6	107.1(5)
N12–Pt1–C19	179.4(2)	C13–N18–Pt1	113.5(5)
N18–Pt1–N1	161.3(3)	C17–N18–Pt1	126.6(6)
C19–Pt1–N1	99.3(3)	C17–N18–C13	119.9(7)
C19–Pt1–N18	99.3(3)	C26–C25–C24	120.8(7)
O3–S1–O2	114.5(3)	C3–C4–C5	120.7(6)
O3–S1–C28	103.0(3)	C23–C22–C21	119.8(6)
O2–S1–C28	103.0(3)	C10–N9–C8	119.1(6)
O1–S1–O3	115.5(3)	C26–C21–C20	119.9(6)
O1–S1–O2	115.2(3)	C22–C21–C26	119.2(6)
O1–S1–C28	103.0(3)	C22–C21–C20	120.8(6)
C15–C16–C17	118.5(7)	N9–C10–C11	123.3(7)
C23–C24–C27	118.7(6)	N18–C13–C11	115.4(7)
C23–C24–C25	119.4(7)	N18–C13–C14	121.1(7)
C25–C24–C27	121.9(7)	C14–C13–C11	123.5(7)
C6–N1–Pt1	112.6(5)	C4–C3–C2	117.9(7)
C2–N1–Pt1	126.9(5)	C19–C20–C21	175.4(7)
C2–N1–C6	120.5(7)	N12–C7–C6	112.1(6)
F2–C27–C24	111.8(8)	N12–C7–C8	118.2(6)
F3–C27–F2	104.7(8)	C8–C7–C6	129.7(6)
F3–C27–F1	106.1(9)	C20–C19–Pt1	179.2(6)
F3–C27–C24	113.8(6)	N12–C11–C10	115.3(6)
F1–C27–F2	106.3(6)	N12–C11–C13	113.0(6)
F1–C27–C24	113.4(7)	C10–C11–C13	131.7(7)
C7–N12–Pt1	119.1(5)	N1–C6–C7	115.8(6)
C7–N12–C11	123.8(6)	N1–C6–C5	120.6(7)
C11–N12–Pt1	117.0(5)	C5–C6–C7	123.6(7)
C25–C26–C21	120.1(7)	N1–C2–C3	121.7(7)
C22–C23–C24	120.7(6)	C13–C14–C15	118.6(7)

F5–C28–S1	111.5(4)	C6–C5–C4	118.7(7)
F5–C28–F6	107.5(6)	N9–C8–C7	120.2(6)
F6–C28–S1	111.6(5)	C16–C15–C14	120.9(7)
F4–C28–S1	111.6(5)	N18–C17–C16	121.0(8)

Photophysical Studies

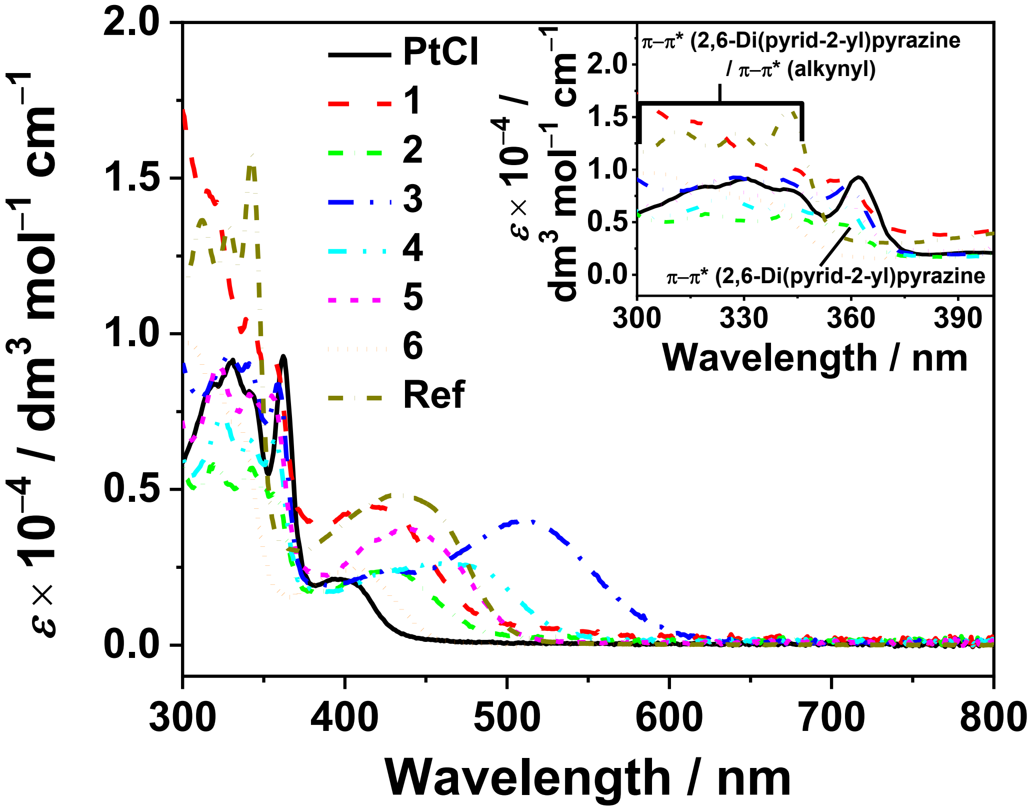

Figure S4. UV–Vis absorption spectra of **PtCl**, **1**–**6** and **Ref** in acetonitrile ([Pt] ~ 10^{-5} M). (Inset) Magnified UV–vis absorption spectra showing the region of 300–400 nm.

Table S13. Electronic absorption data of platinum(II) 2,6-(dipyrid-2-yl)pyrazine complexes at 298 K

Complex	λ_{abs}/nm (ε/mol^{-1}dm^3 cm^{-1})
**PtCl**	331 (9160), 361 (9110), 400 (2090)
**1**	316 (14600), 340 (10300), 357 (8980), 421 (4430), 531 sh (540)
**2**	321 (5770), 343 (5690), 357 (4800), 429 (2430), 544 sh (240)
**3**	327 (9260), 341 (9050), 359 (8380), 426 (2390), 511 (4020)
**4**	324 (7300), 341 (6610), 358 (6580), 460 (2610)
**5**	322 (8980), 341 (8060), 355 (8010), 439 (3750)
**6**	307 (9640), 322 (8240), 336 (7000), 407 (2460)
**Ref**	311 (13600), 328 (13400), 343 (15700), 440 (4780)

Variable-Temperature UV–vis Absorption Studies

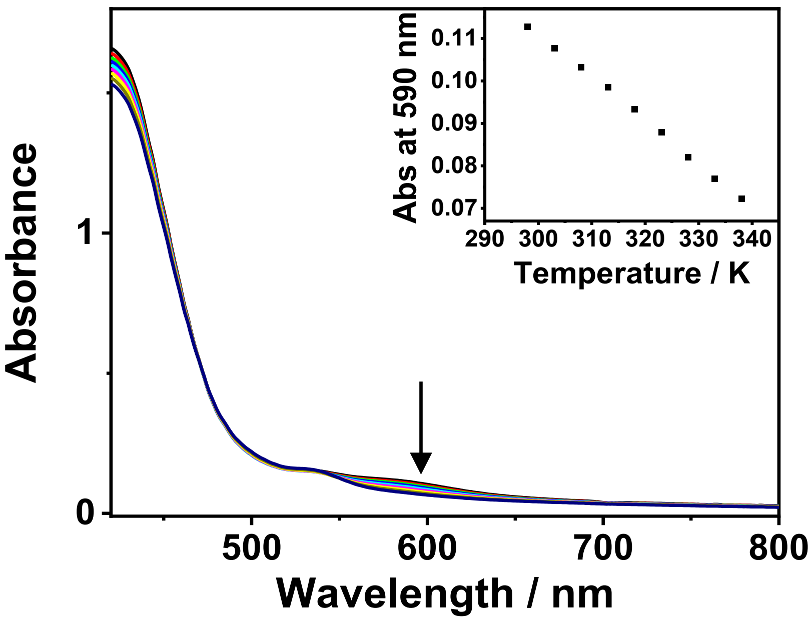

Figure S5. UV–Vis absorption spectra of **1** in acetonitrile ([Pt] = 712 μM) upon increasing temperature from 298 to 338 K. (Inset) A plot of absorbance at 590 nm against temperature.

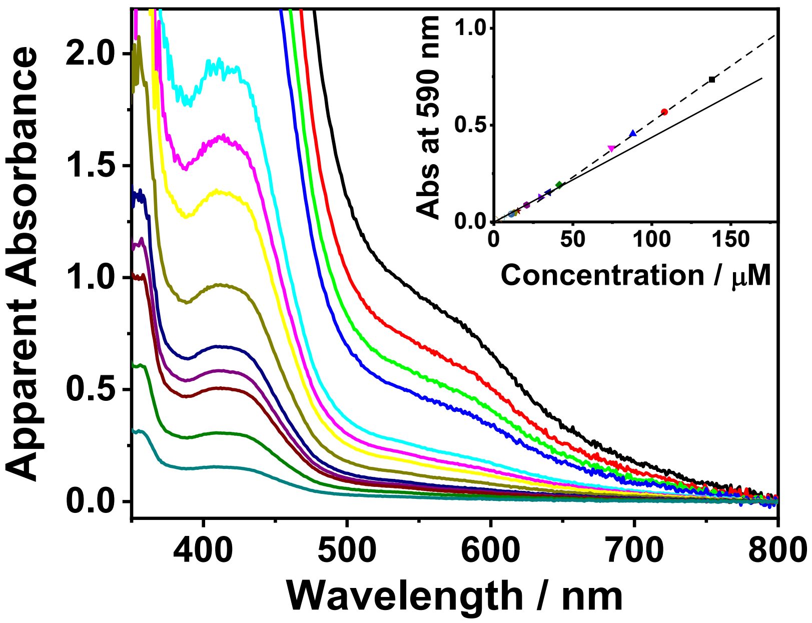

Figure S6. UV–Vis absorption spectra of **1** in acetonitrile on increasing concentration from 3 to 1610 μM. (Inset) A plot of apparent absorbance at 590 nm against concentration. The apparent absorbance values have been obtained by correcting to a 1-cm path length equivalence.

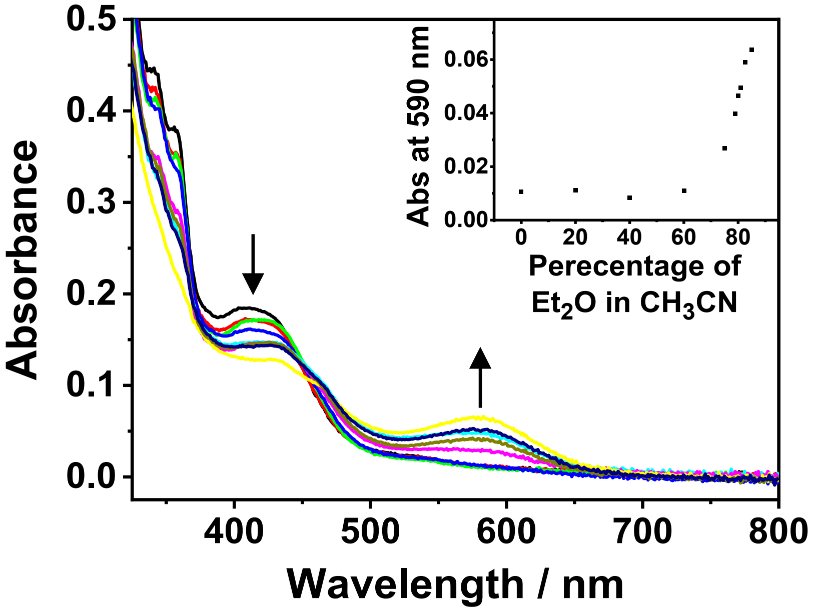

Figure S7. UV–Vis absorption spectral traces of **1** in acetonitrile ([Pt] = 38 μM) with increasing diethyl ether content from 0 to 85 % (v/v). (Inset) A plot of absorbance at 580 nm against diethyl ether content in acetonitrile.

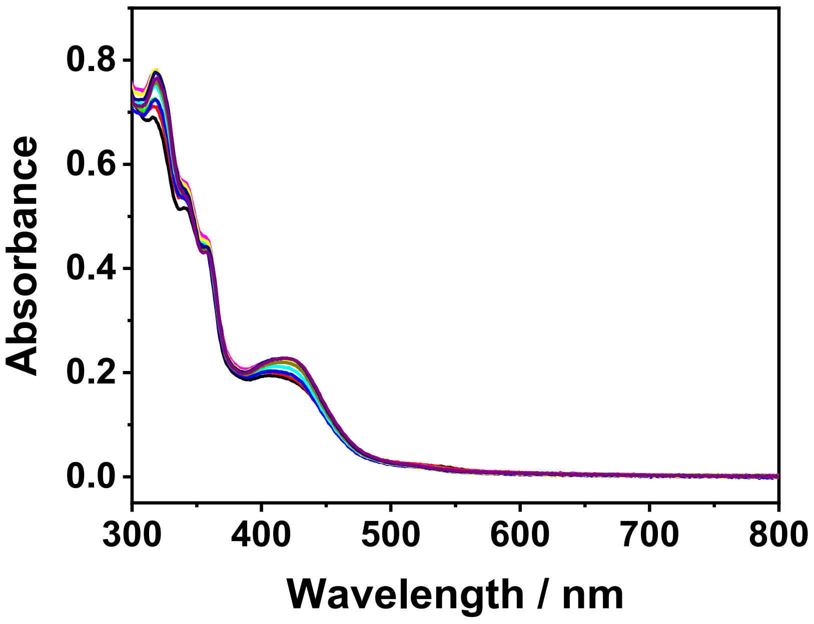

Figure S8. UV–Vis absorption spectral traces of **1** in acetonitrile ([Pt] = 39 μM) with increasing methanol content from 0 to 90 %.

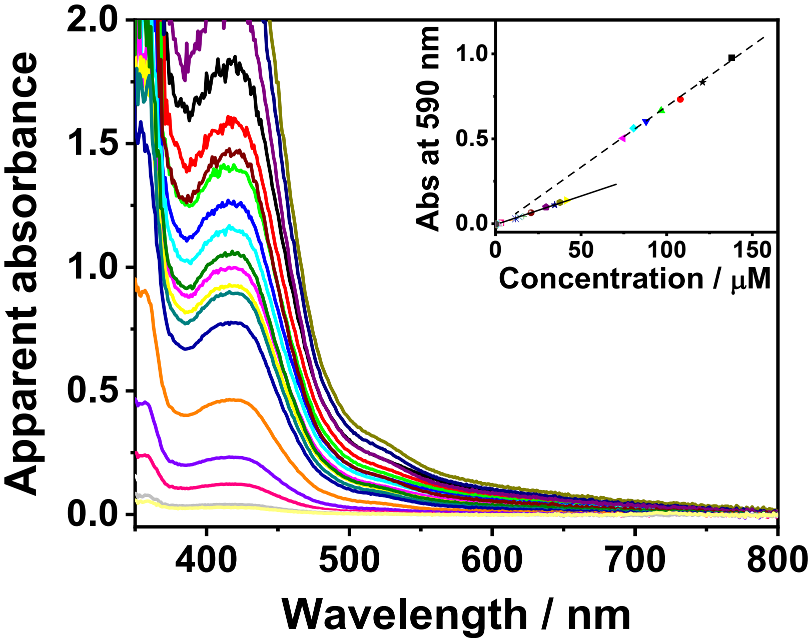

Figure S9. UV–Vis absorption spectra of **1** in acetonitrile–methanol mixture (4:1, v/v) on increasing concentration from 3 to 1210 μM. (Inset) A plot of apparent absorbance at 590 nm against concentration. The apparent absorbance values have been obtained by correcting to a 1-cm path length equivalence.

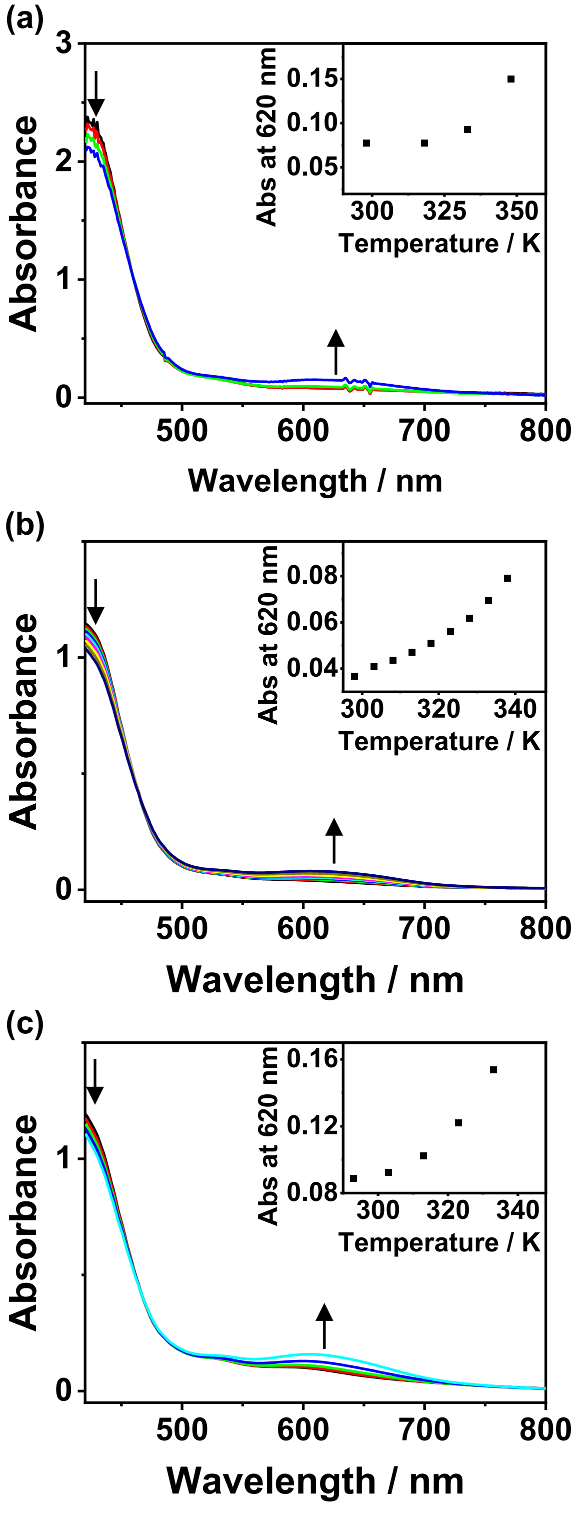

Figure S10. (a) UV–Vis absorption spectra of **1** in acetonitrile–methanol (4:1, v/v) ([Pt] = 969 μM), (b) acetonitrile–isopropanol (4:1, v/v) ([Pt] = 694 μM) and (c) acetonitrile–*n*-butanol (4:1, v/v) ([Pt] = 1040 μM) upon increasing temperature from 298 to 338 K. (Inset) The plots of absorbance at 620 nm against temperature.

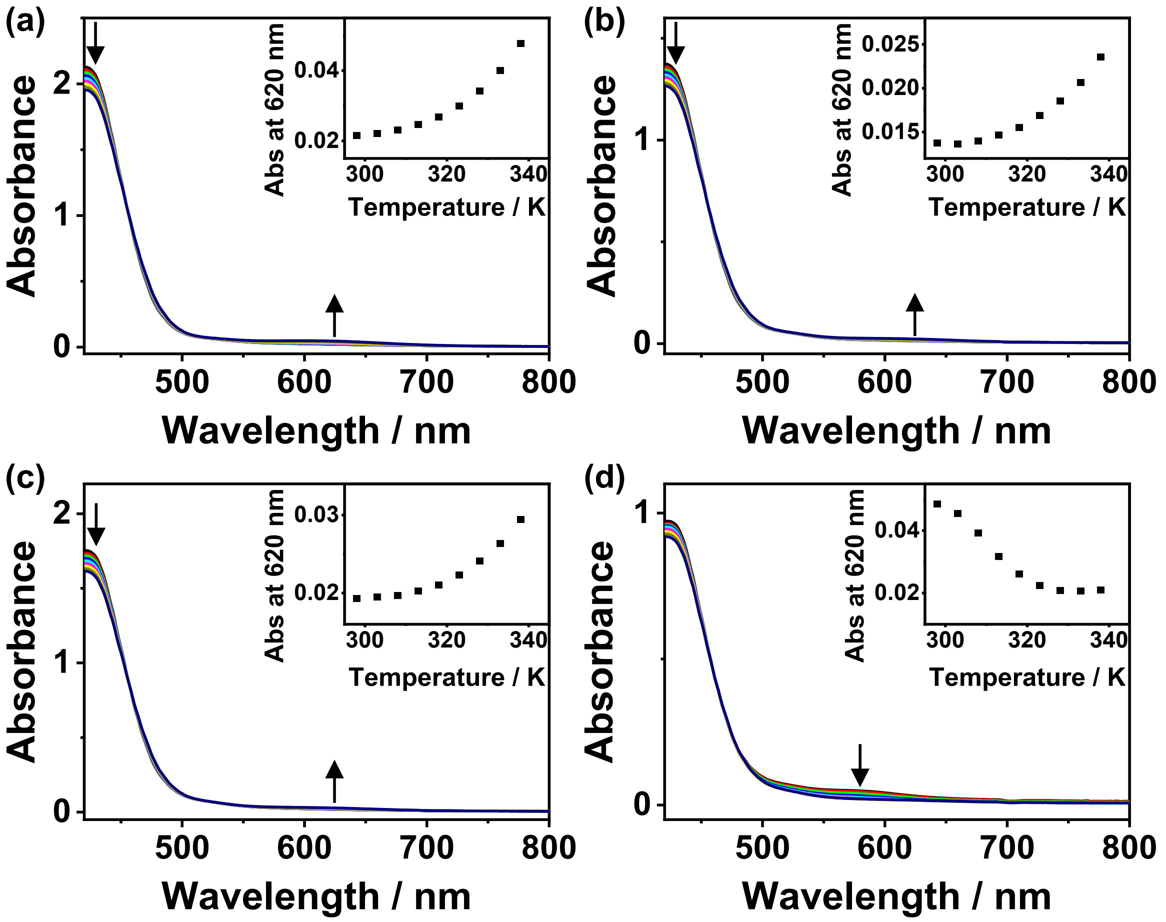

Figure S11. UV−Vis absorption spectra of **1** in (a) acetonitrile–ethanol (3:2, v/v) ([Pt] = 690 μM), (b) acetonitrile–ethanol (2:3, v/v) ([Pt] = 690 μM), (c) acetonitrile–ethanol (1:4, v/v) ([Pt] = 708 μM) upon increasing temperature from 298 to 338 K. (Inset) The plots of absorbance at 620 nm against temperature. (d) UV−Vis absorption spectra of **1** in ethanol ([Pt] = 664 μM) upon increasing temperature from 298 to 338 K. (Inset) A plot of absorbance at 590 nm against temperature.

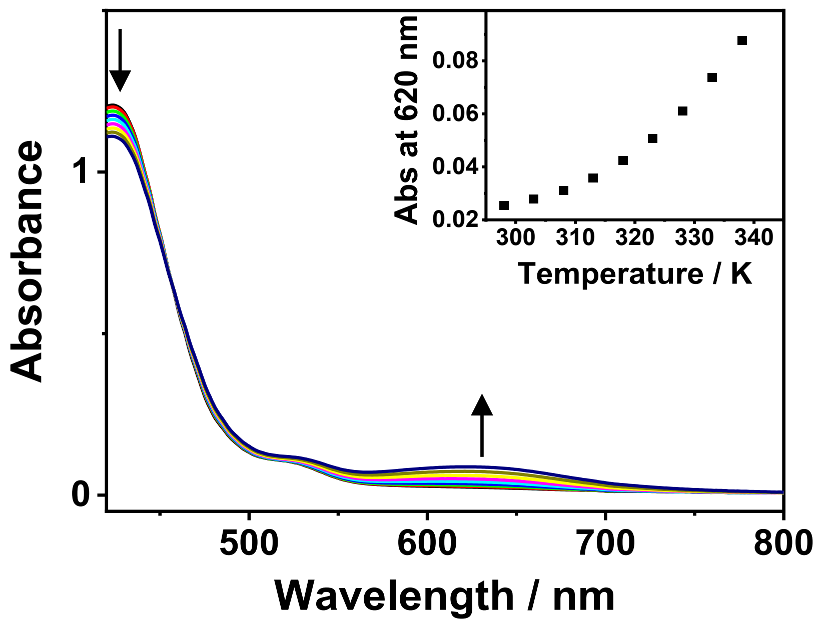

Figure S12. UV–Vis absorption spectra of **2** in acetonitrile–ethanol mixture (4:1, v/v) ([Pt] = 652 μM) upon increasing temperature from 298 to 338 K. (Inset) A plot of absorbance at 620 nm against temperature.

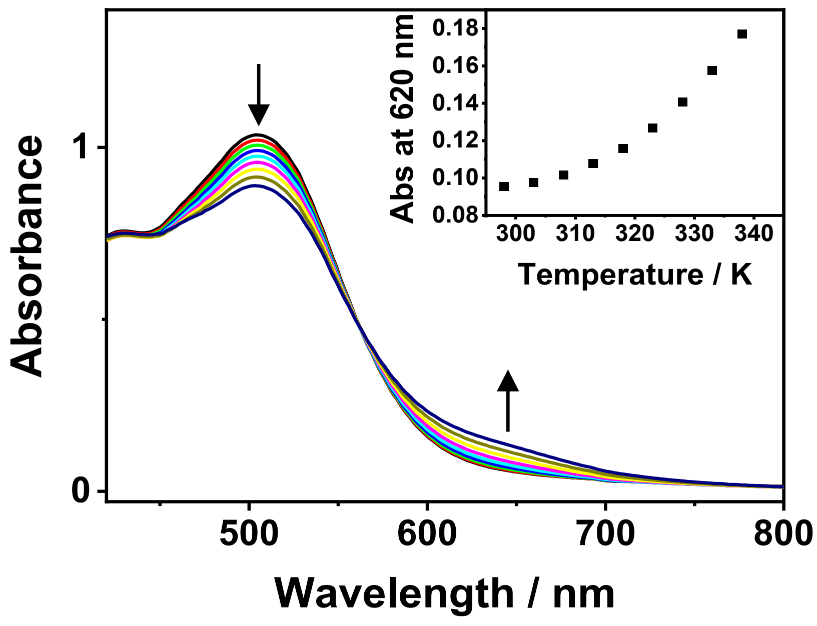

Figure S13. UV–Vis absorption spectra of **3** in acetonitrile–ethanol mixture (4:1, v/v) ([Pt] = 121 μM) upon increasing temperature from 298 to 338 K. (Inset) A plot of absorbance at 620 nm against temperature.

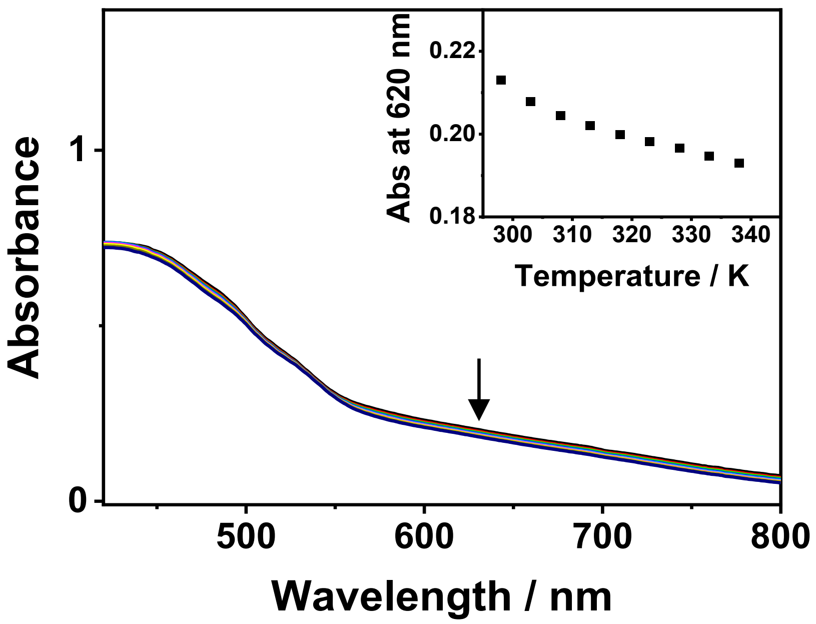

Figure S14. UV–Vis absorption spectra of **4** in acetonitrile–ethanol mixture (4:1, v/v) ([Pt] = 121 μM) upon increasing temperature from 298 to 338 K. (Inset) A plot of absorbance at 620 nm against temperature.

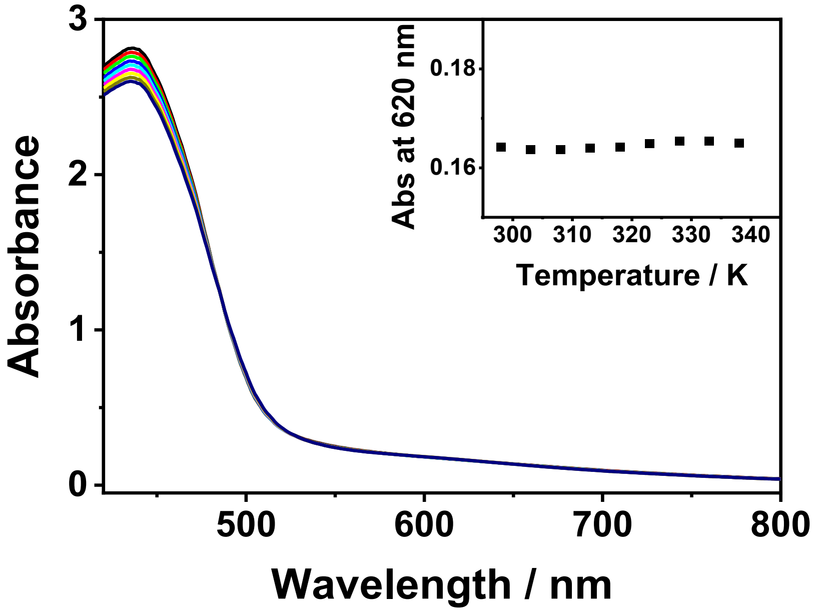

Figure S15. UV–Vis absorption spectra of **5** in acetonitrile–ethanol mixture (4:1, v/v) ([Pt] = 652 μM) upon increasing temperature from 298 to 338 K. (Inset) A plot of absorbance at 620 nm against temperature.

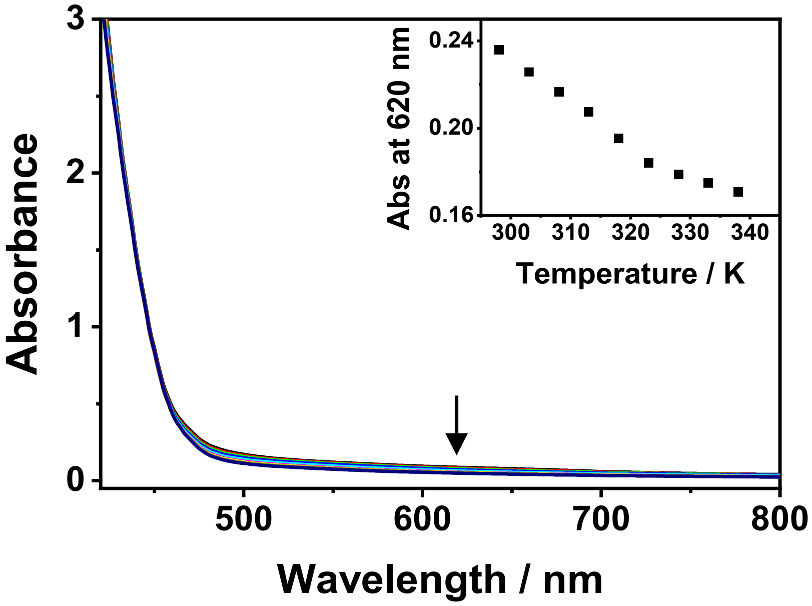

Figure S16. UV–Vis absorption spectra of **6** in acetonitrile–ethanol mixture (4:1, v/v) ([Pt] = 742 μM) upon increasing temperature from 298 to 338 K. (Inset) A plot of absorbance at 480 nm against temperature.

Variable-Temperature ^{1}H NMR Spectra

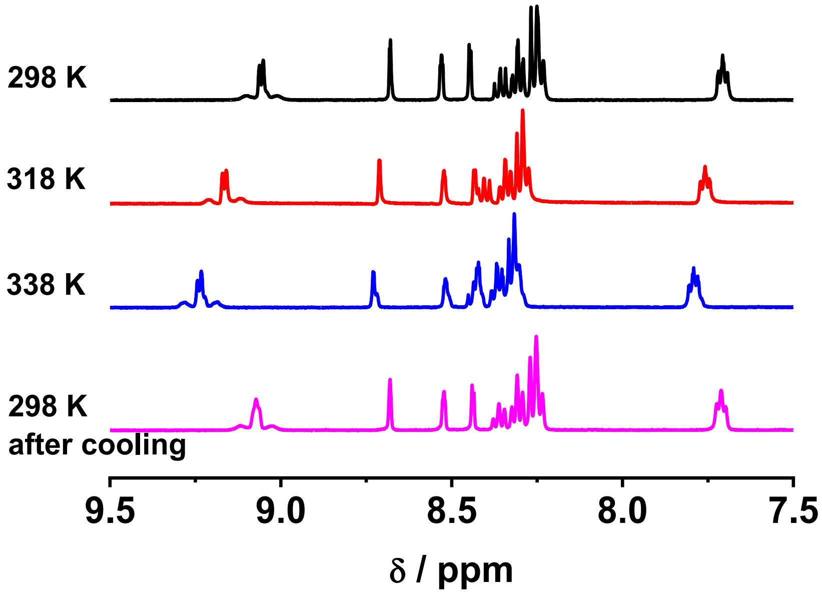

Figure S17. Partial ^{1}H NMR spectra of **6** in CD_3CN–CD_3OD (4:1, v/v) in the aromatic region ([Pt] = 2640 μM) upon increasing the temperature from 298 K to 338 K.

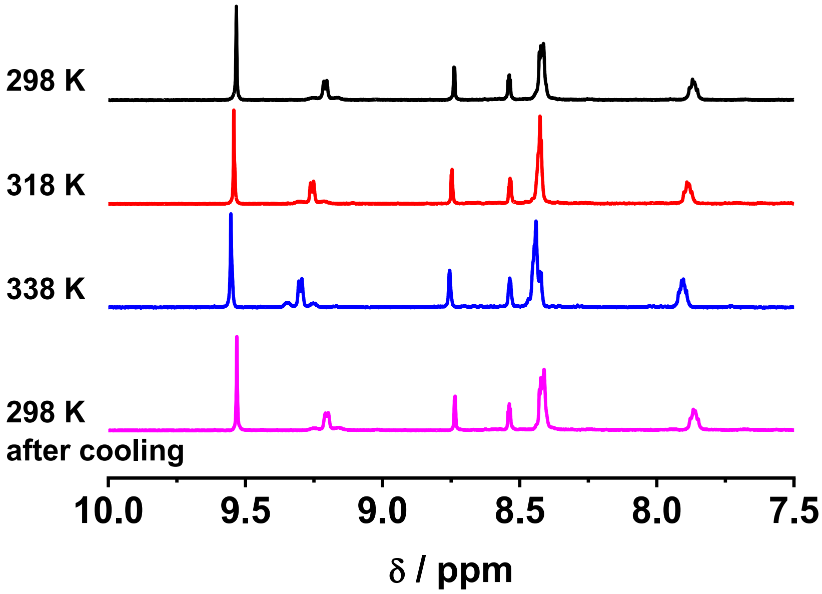

Figure S18. Partial ^{1}H NMR spectra of **1** in CD_3CN in the aromatic region ([Pt] = 2310 μM) upon increasing the temperature from 298 to 338 K.

UV–Vis Absorption Studies upon Addition of Acid and Base

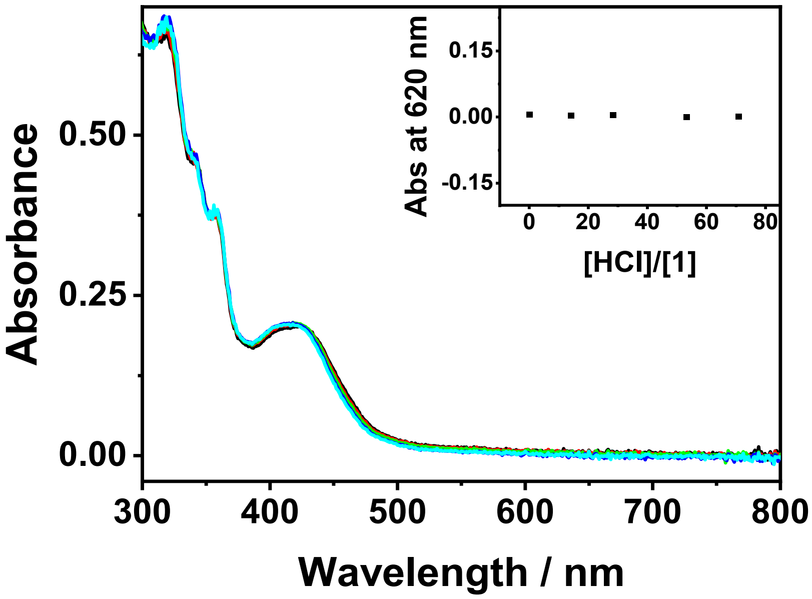

Figure S19. UV–Vis absorption spectra of **1** in acetonitrile–ethanol mixture (4:1, v/v) ([Pt] = 36 μM) upon addition of hydrochloric acid (HCl). (Inset) A plot of absorbance at 620 nm against the concentration ratio, [HCl]/[**1**].

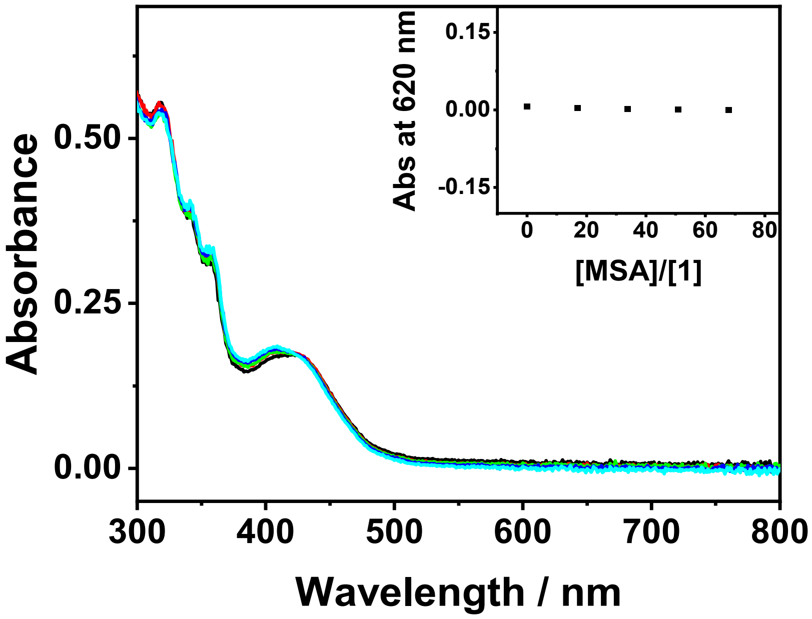

Figure S20. UV–Vis absorption spectra of **1** in acetonitrile–ethanol mixture (4:1, v/v) ([Pt] = 28 μM) upon addition of methanesulfonic acid (MSA). (Inset) A plot of absorbance at 620 nm against the concentration ratio, [MSA]/[**1**].

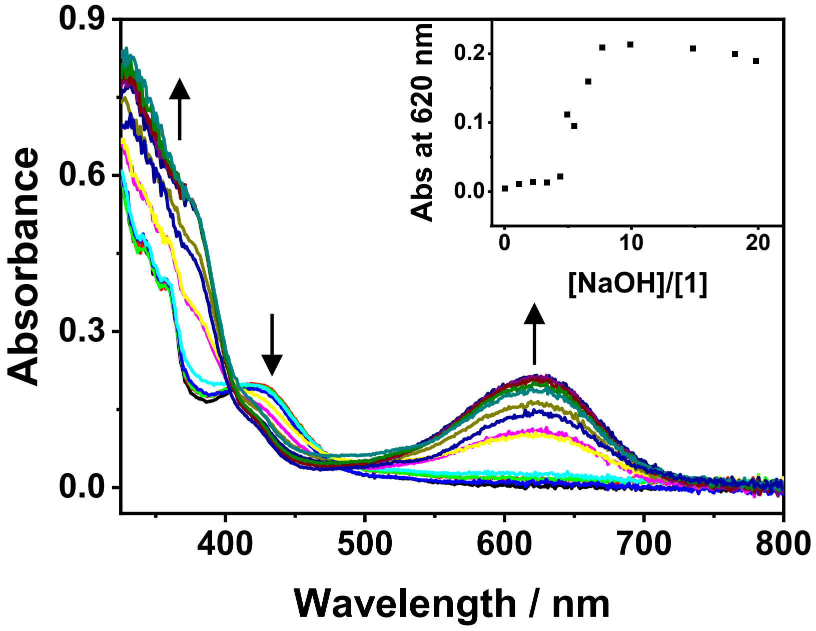

Figure S21. UV–Vis absorption spectra of **1** in acetonitrile–ethanol mixture (4:1, v/v) ([Pt] = 36 μM) upon addition of sodium hydroxide. (Inset) A plot of absorbance at 620 nm against the concentration ratio, [NaOH]/[**1**].

Variable-Temperature FT-IR Spectra

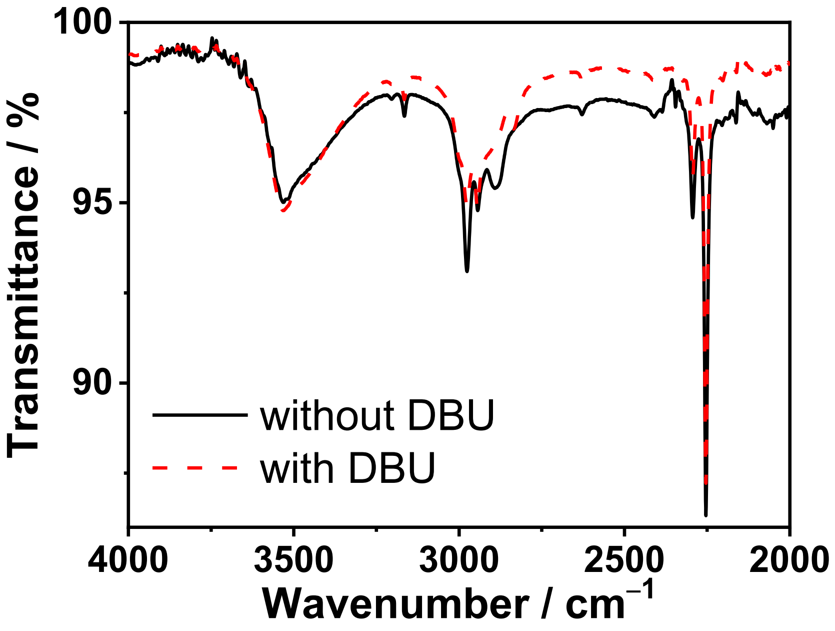

Figure S22. FT-IR spectra of the blank sample containing the solvent mixture of acetonitrile–ethanol (4:1, v/v) before and after addition of DBU.

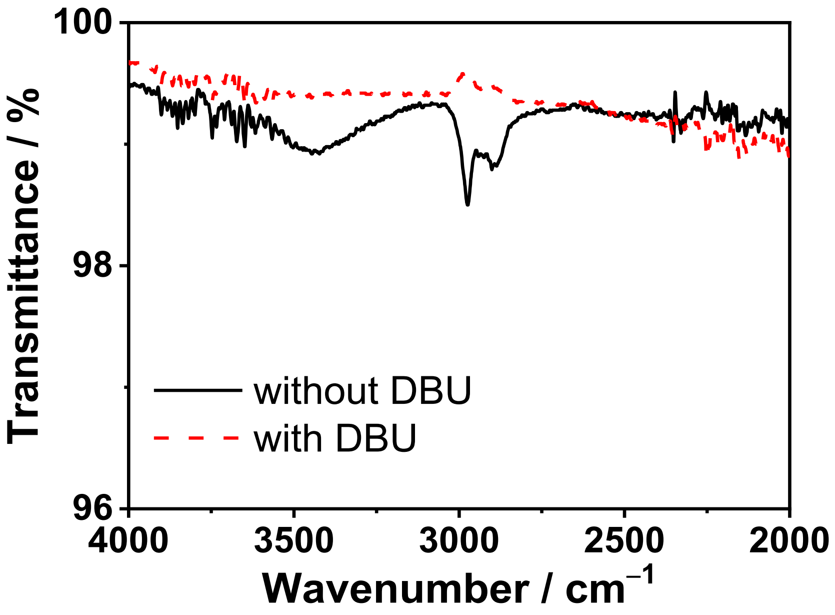

Figure S23. FT-IR spectra of **1** in acetonitrile–ethanol mixture (4:1, v/v) ([Pt] = 481 μM) before and after addition of DBU.

^{1}H–^{1}H NOESY NMR spectra

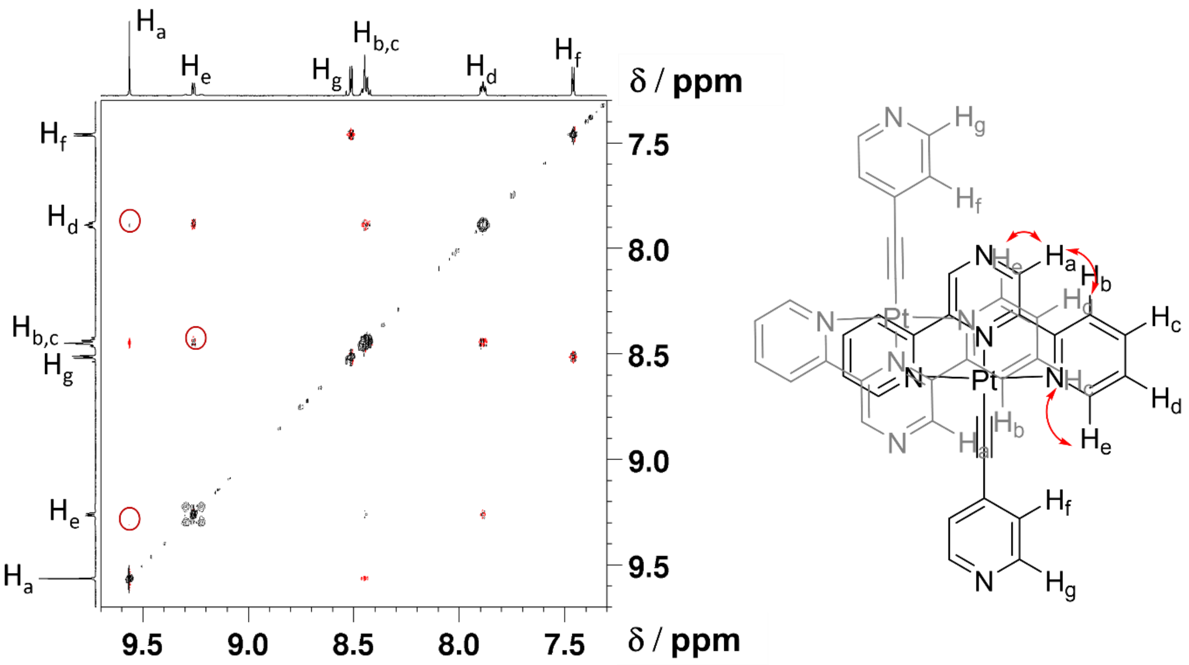

Figure S24. Partial ^{1}H–^{1}H NOESY NMR spectra of **2** in CD_3CN–CD_3OD (4:1, v/v) ([Pt] = 3890 μM) at 298 K and its plausible association mode.

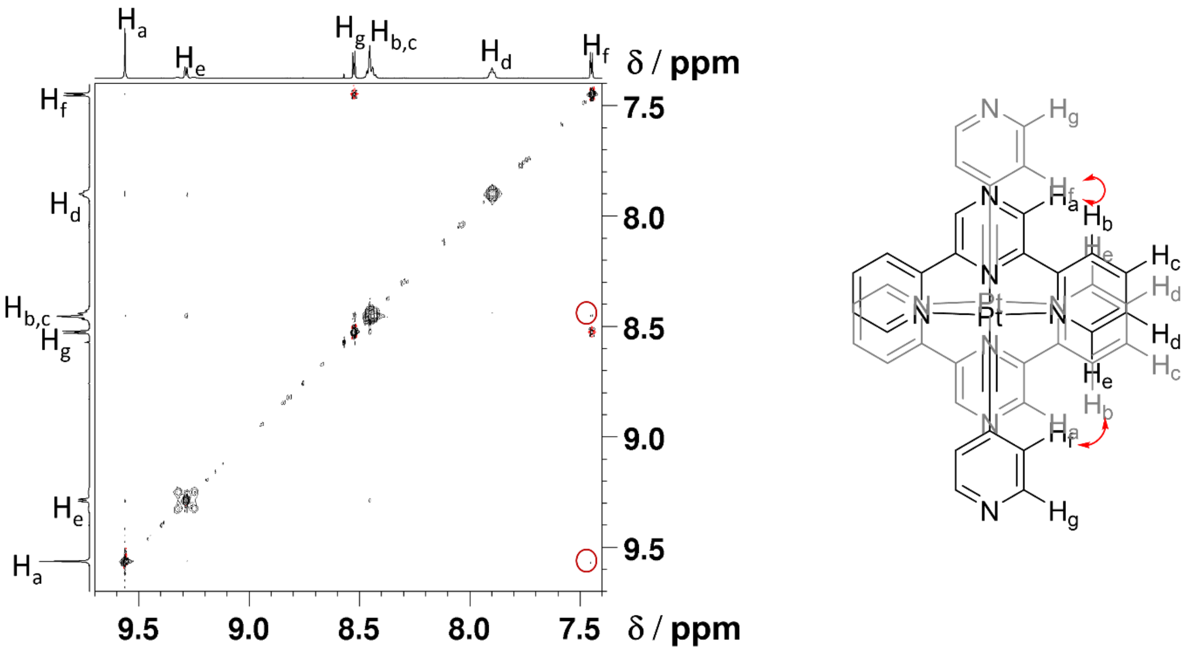

Figure S25. Partial ^{1}H–^{1}H NOESY NMR spectra of **2** in CD_3CN–CD_3OD (4:1, v/v) ([Pt] = 3890 μM) at 333 K and its plausible association mode.

Computational Results

Table S14. Density (ρ), dielectric constant (ε), isothermal compressibility (κ_T) and thermal coefficient of volume expansion at constant pressure (α_P) of acetonitrile and ethanol at 298 K.

		ρ / kg·m^{-3}	ε	κ_T / bar^{-1}	α_P / K^{-1}
acetonitrile	MD	804	35.0	0.000145	0.001264
	experiment	786	37.5	0.000120	0.001397
ethanol	MD	793	14.4	0.000138	0.00160
	experiment	789	24.5	0.000111	0.00109

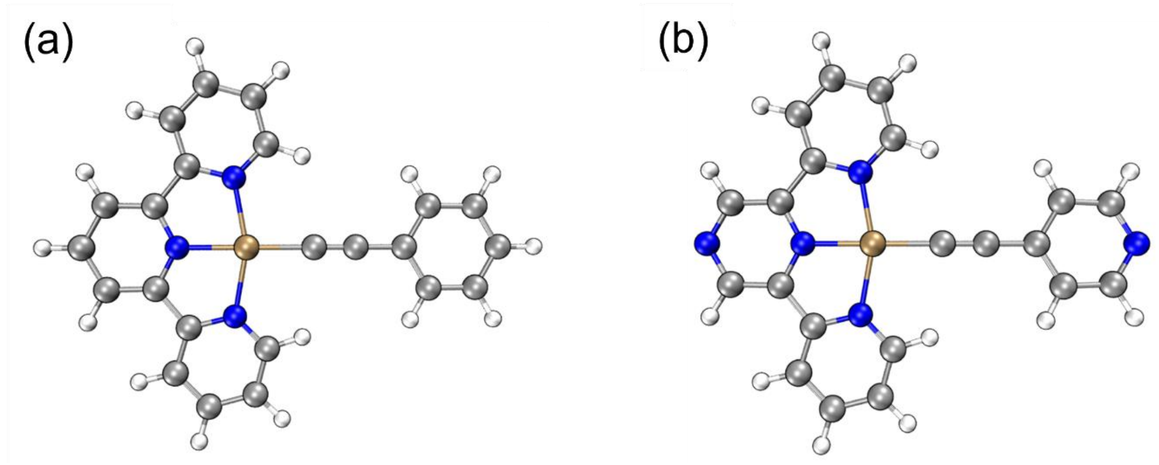

Figure S26. Optimized S_0 structure of complex cations of (a) **Ref** and (b) **2**.

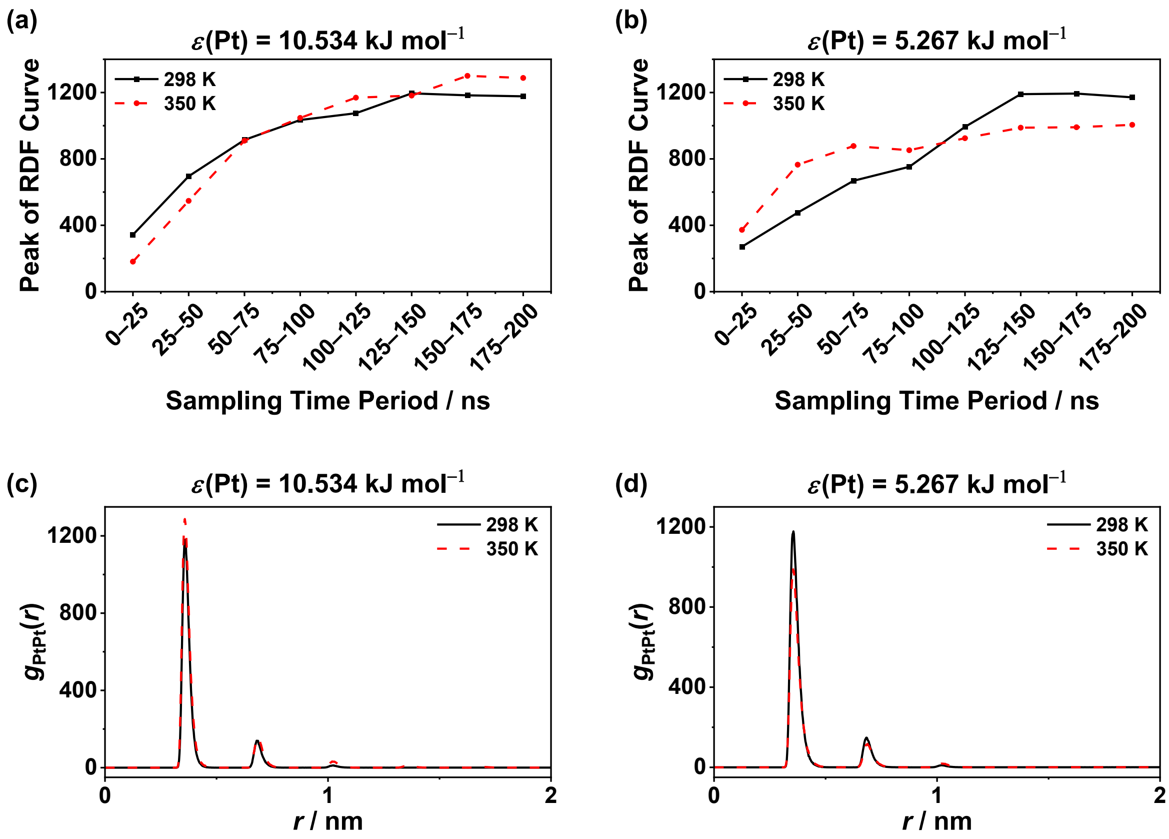

Figure S27. MD simulations for the control complex, **Ref**, in acetonitrile–ethanol mixture. The intensity of the first $g_{\mathrm{PtPt}}(r)$ peak as a function of simulation time period using (a) ε(Pt) = 10.534 kJ mol^{-1} and (b) ε(Pt) = 5.267 kJ mol^{-1} are presented. Curves of $g_{\mathrm{PtPt}}(r)$ in (c) and (d) are prepared using the trajectory in the range of 150–200 ns, where the intensity of the first $g_{\mathrm{PtPt}}(r)$ peak reaches equilibrium.

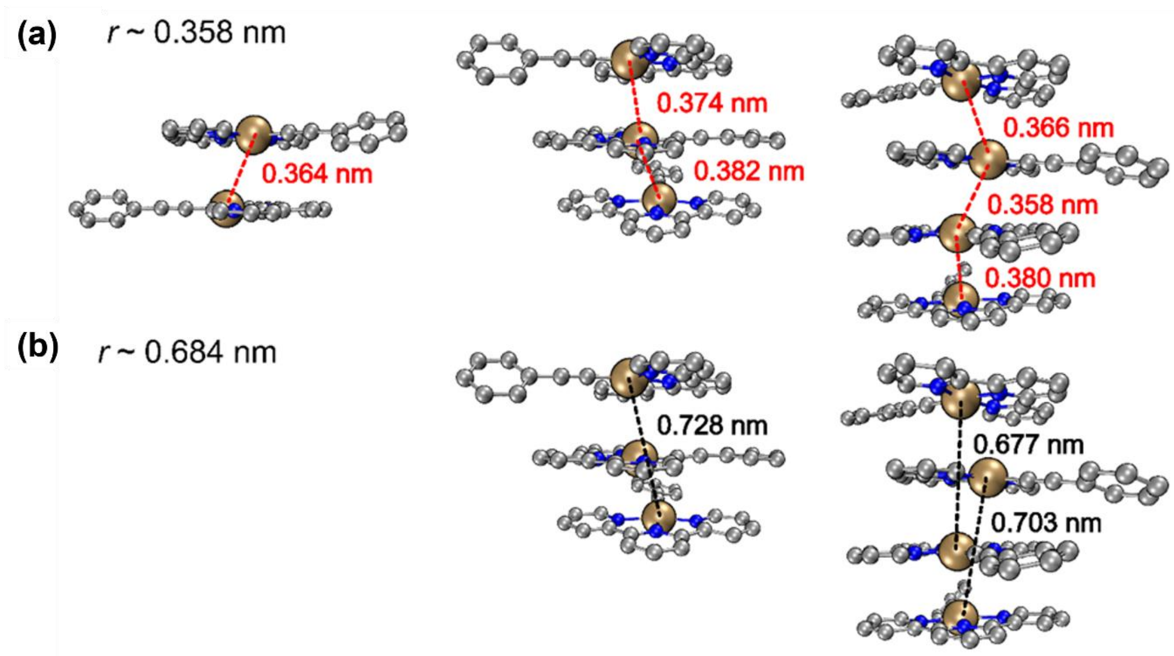

Figure S28. Computed assembled structures corresponding to the first peak at (a) $r \sim 0.358$ nm and the second peak at (b) $r \sim 0.684$ nm.

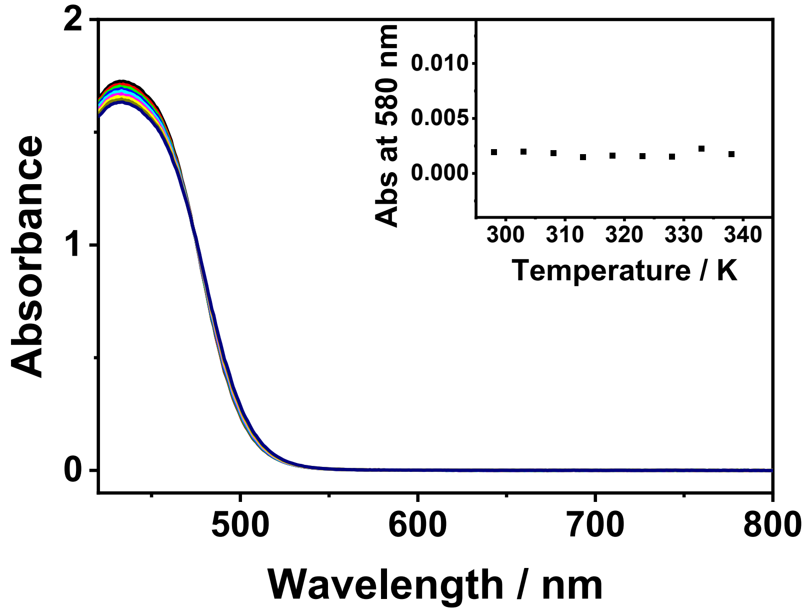

Figure S29. UV–Vis absorption spectra of **Ref** in acetonitrile–ethanol (4:1, v/v) ([Pt] = 389 μM) upon increasing temperature from 298 to 338 K. (Inset) A plot of absorbance at 580 nm against temperature.

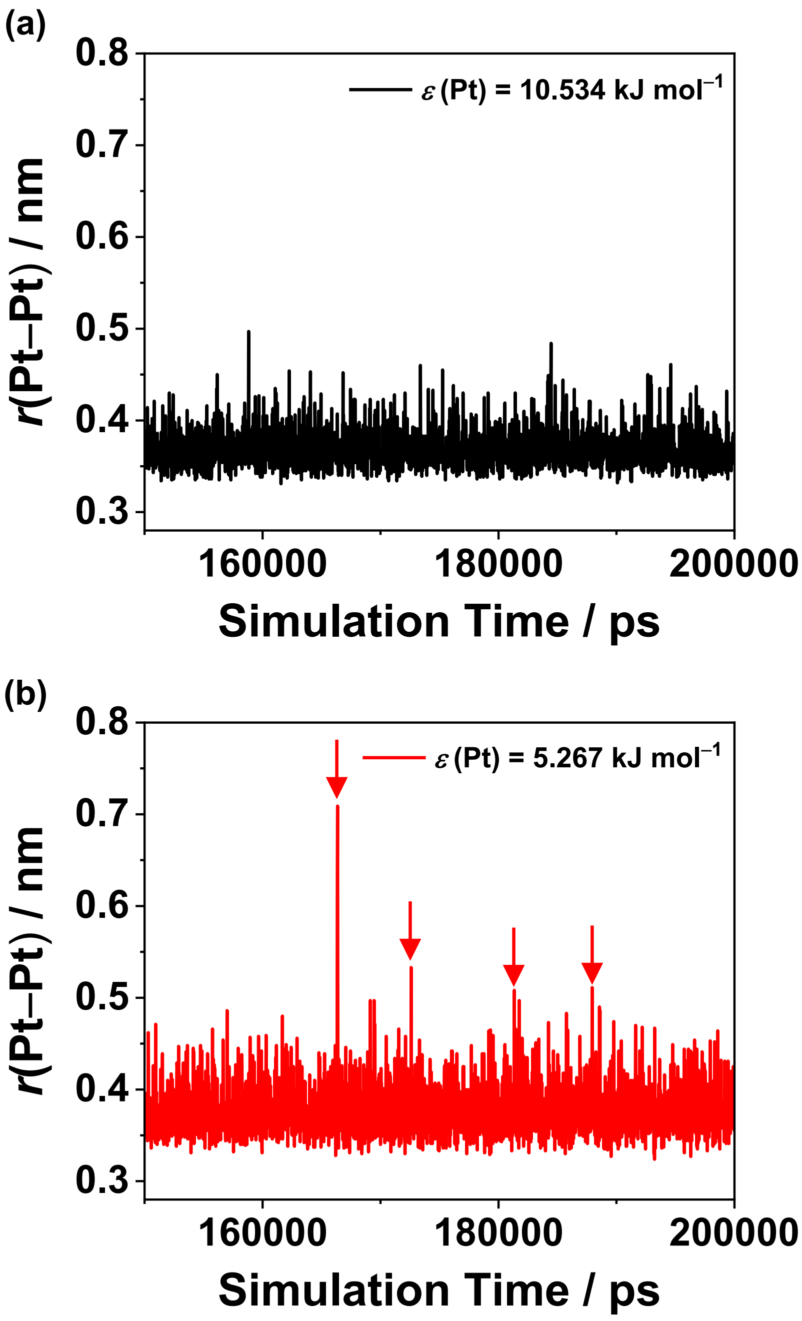

Figure S30. Pt···Pt distance as a function of simulation time at 350 K using (a) ε(Pt) = 10.534 kJ mol^{-1} and (b) ε(Pt) = 5.267 kJ mol^{-1}. Temporary disruptions of Pt···Pt interactions (r(Pt···Pt) > 0.5 nm) are indicated.

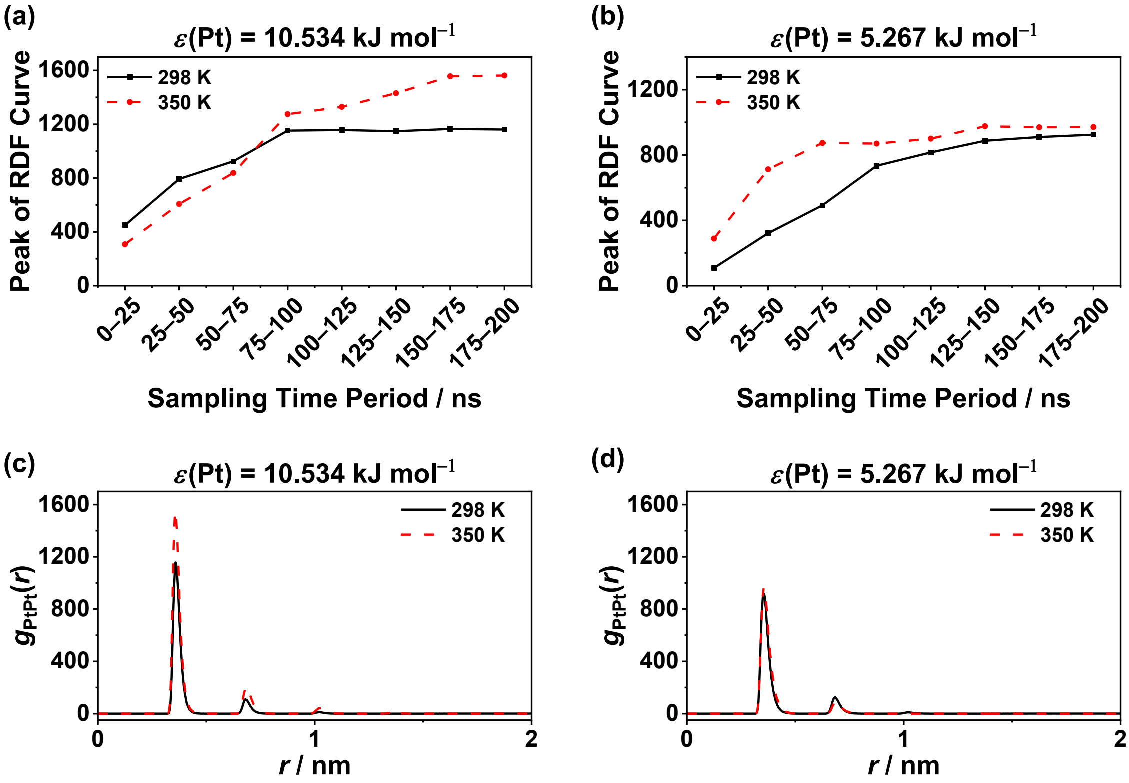

Figure S31. Replicated MD simulations for the control complex, **Ref**, in acetonitrile–ethanol mixture with a different initial configuration. The intensity of the first $g_{\mathrm{PtPt}}(r)$ peak as a function of simulation time period using (a) ε(Pt) = 10.534 kJ mol^{-1} and (b) ε(Pt) = 5.267 kJ mol^{-1} are presented. Curves of $g_{\mathrm{PtPt}}(r)$ in (c) and (d) are prepared using the trajectory in the range of 150–200 ns, where the intensity of the first $g_{\mathrm{PtPt}}(r)$ peak reaches equilibrium.

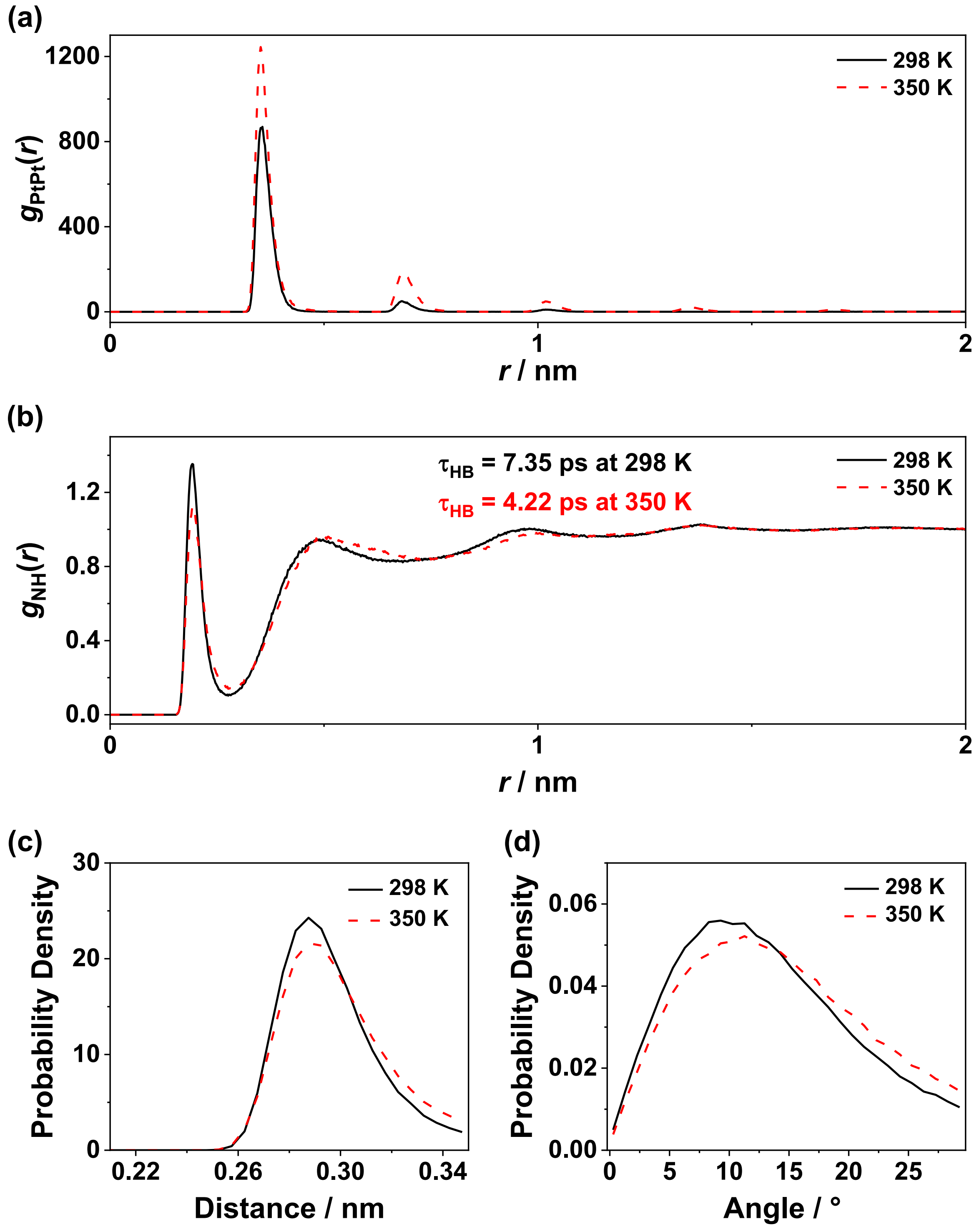

Figure S32. Replicated MD simulations for **2** in acetonitrile–ethanol mixture with a different initial configuration. (a) $g_{\mathrm{PtPt}}(r)$ curves at 298 K and 350 K prepared using the trajectory in the range of 150–200 ns. (b) Radial distribution function $g_{\mathrm{NH}}(r)$ and distributions of (c) N···O distance and (d) N···O–H angle for hydrogen bonds between **2** and ethanol.

Table S15. Cartesian coordinates of the optimized S_0 structure of **2**

C	−2.943497	1.179906	0.003630	H	−1.786982	−5.702249	−0.020798
C	−2.943526	−1.179932	−0.003517	H	1.259149	−2.652913	−0.008840
C	−4.339587	−1.151692	−0.003242	H	0.643238	−5.080176	−0.018484
C	−4.339560	1.151716	0.003599	Pt	−0.339537	−0.000006	−0.000030
C	−2.023697	2.328248	0.007242	N	−0.691146	2.009182	0.005775
C	0.226240	2.980964	0.009531	N	−2.312584	−0.000020	0.000002
C	−0.132381	4.323714	0.014829	N	−0.691173	−2.009194	−0.005978
C	−1.477797	4.662979	0.016283	C	1.618814	0.000148	0.000293
C	−2.434805	3.651462	0.012414	C	2.845502	0.000372	0.000426
H	−4.927444	−2.064042	−0.005940	C	4.267394	0.000101	0.000340
H	−4.927391	2.064088	0.006387	C	4.999108	−1.194926	0.086920
H	1.259179	2.652921	0.008319	C	6.386351	1.135248	−0.082653
H	0.643210	5.080190	0.017889	H	4.487175	−2.148724	0.156515
H	−1.786996	5.702219	0.020543	H	6.965023	2.054746	−0.149649
H	−3.491018	3.891966	0.013712	N	−5.010812	0.000020	0.000238
C	−2.023724	−2.328279	−0.007284	C	4.999330	1.194985	−0.086559
C	−2.434809	−3.651503	−0.012462	H	4.487521	2.148861	−0.156042
C	−1.477786	−4.663009	−0.016526	C	6.386150	−1.135461	0.082564
H	−3.491017	−3.892055	−0.013620	H	6.964654	−2.055083	0.149315
C	0.226219	−2.980966	−0.009922	N	7.088027	−0.000182	−0.000159
C	−0.132375	−4.323722	−0.015254				

Table S16. Cartesian coordinates of the optimized S_0 structure of **Ref**

C	2.951154	−1.187066	0.002709	H	−1.267289	2.636894	−0.008047
C	2.951137	1.187086	−0.002787	H	−0.665836	5.069801	−0.015680
C	4.342026	1.214454	−0.002885	Pt	0.343327	−0.000001	0.000016
C	4.342044	−1.214417	0.002676	N	0.686561	−2.005725	0.005016
C	2.016776	−2.328485	0.005801	N	2.326013	0.000004	−0.000010
C	−0.236774	−2.973113	0.008649	N	0.686530	2.005722	−0.005016
C	0.113487	−4.317047	0.012985	C	−1.616858	0.000009	0.000040
C	1.458002	−4.662218	0.013583	C	−2.844452	0.000015	0.000025
C	2.418816	−3.655260	0.009928	C	−4.269838	0.000009	0.000006
H	4.886426	2.150882	−0.005061	C	−4.986863	1.207801	0.068926
H	4.886460	−2.150836	0.004781	C	−6.376342	−1.203818	−0.068930
H	−1.267255	−2.636911	0.008130	H	−4.439978	2.144365	0.122745
H	−0.665779	−5.069815	0.015898	C	−7.076589	−0.000032	−0.000090
H	1.762564	−5.702968	0.016983	H	−6.916133	−2.144759	−0.122835
H	3.473747	−3.901108	0.010558	H	−8.162447	−0.000073	−0.000132
C	2.016744	2.328493	−0.005815	C	5.026038	0.000023	−0.000137
C	2.418772	3.655272	−0.009867	H	6.110305	0.000031	−0.000194
C	1.457951	4.662225	−0.013432	C	−4.986821	−1.207816	−0.068958
H	3.473702	3.901126	−0.010488	H	−4.439884	−2.144354	−0.122739
C	−0.236810	2.973106	−0.008569	C	−6.376400	1.203759	0.068802
C	0.113439	4.317042	−0.012830	H	−6.916196	2.144700	0.122672
H	1.762507	5.702977	−0.016768				

Electron Microscopy

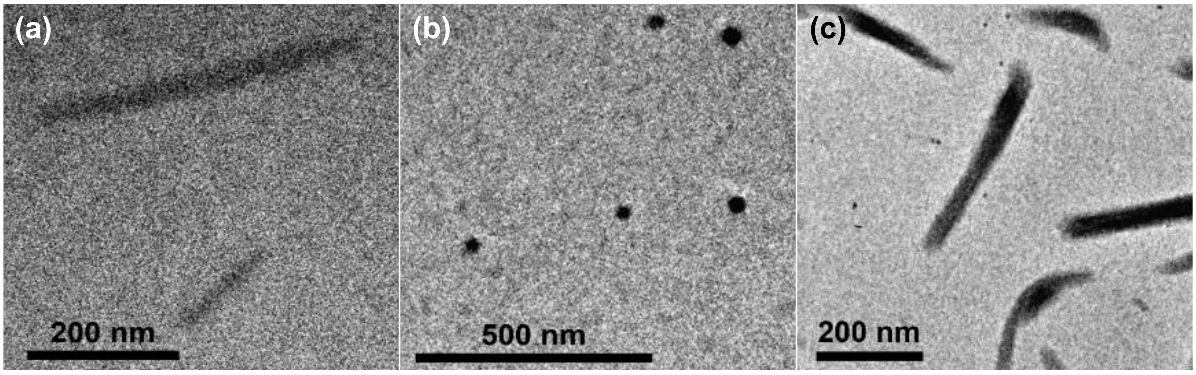

Figure S33. TEM images of **3** in acetonitrile–ethanol mixture (4:1, v/v) ([Pt] ~ 10^{-4} M) (a) without, (b) with heat treatment and (c) after cooling.

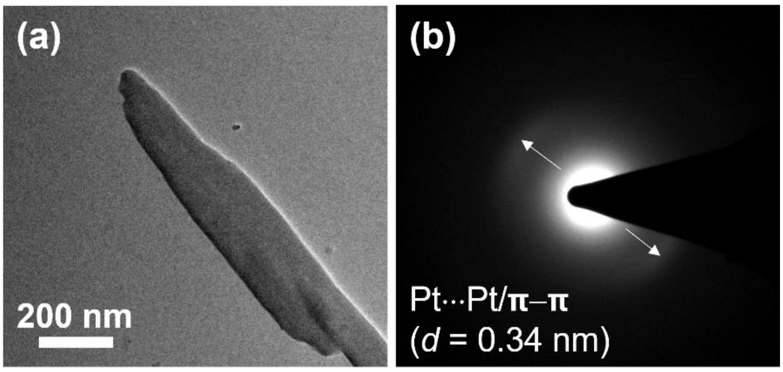

Figure S34. (a) TEM image and (b) SAED pattern of **2** in acetonitrile–ethanol mixture (4:1, v/v) ([Pt] ~ 10^{-4} M) without heat treatment.

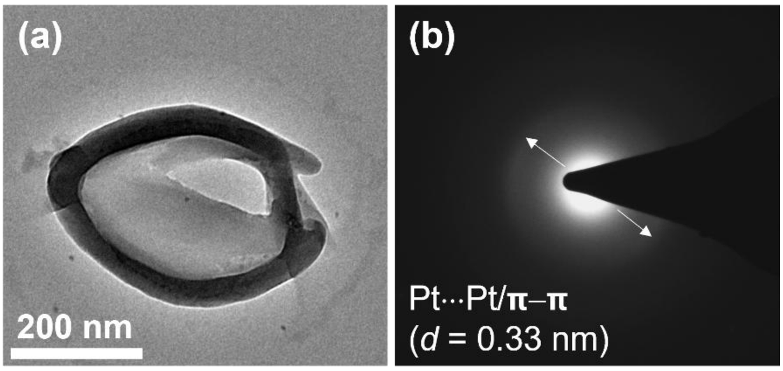

Figure S35. (a) TEM image and (b) SAED pattern of **2** in acetonitrile–ethanol mixture (4:1, v/v) ([Pt] ~ 10^{-4} M) with heat treatment

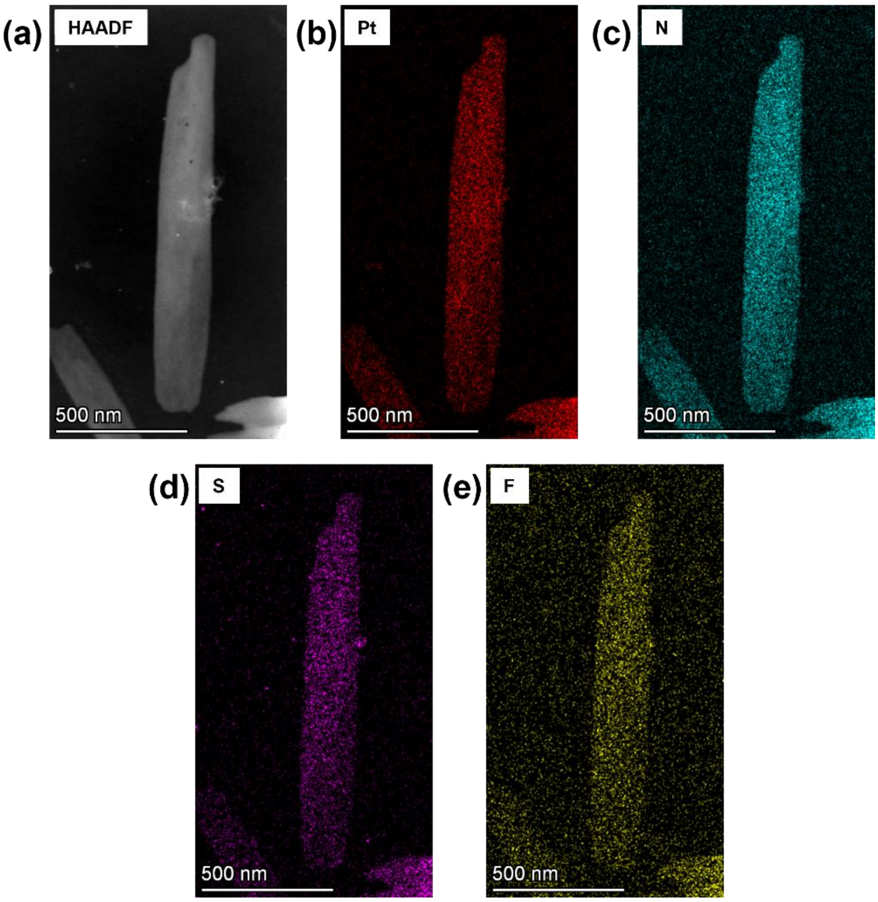

Figure S36. (a) HAADF-STEM and EDX elemental mapping images of (b) Pt, (c) N, (d) S and (e) F of **2** in acetonitrile–ethanol mixture (4:1, v/v) ([Pt] ~ 10^{-4} M) without heat treatment.

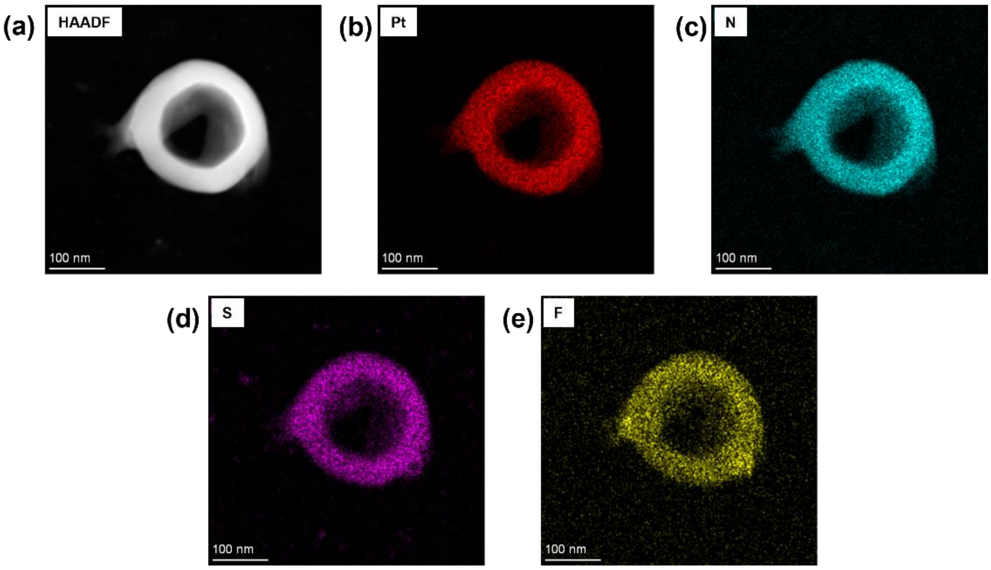

Figure S37. (a) HAADF-STEM and EDX elemental mapping images of (b) Pt, (c) N, (d) S and (e) F of **2** in acetonitrile–ethanol mixture (4:1, v/v) ([Pt] ~ 10^{-4} M) with heat treatment.

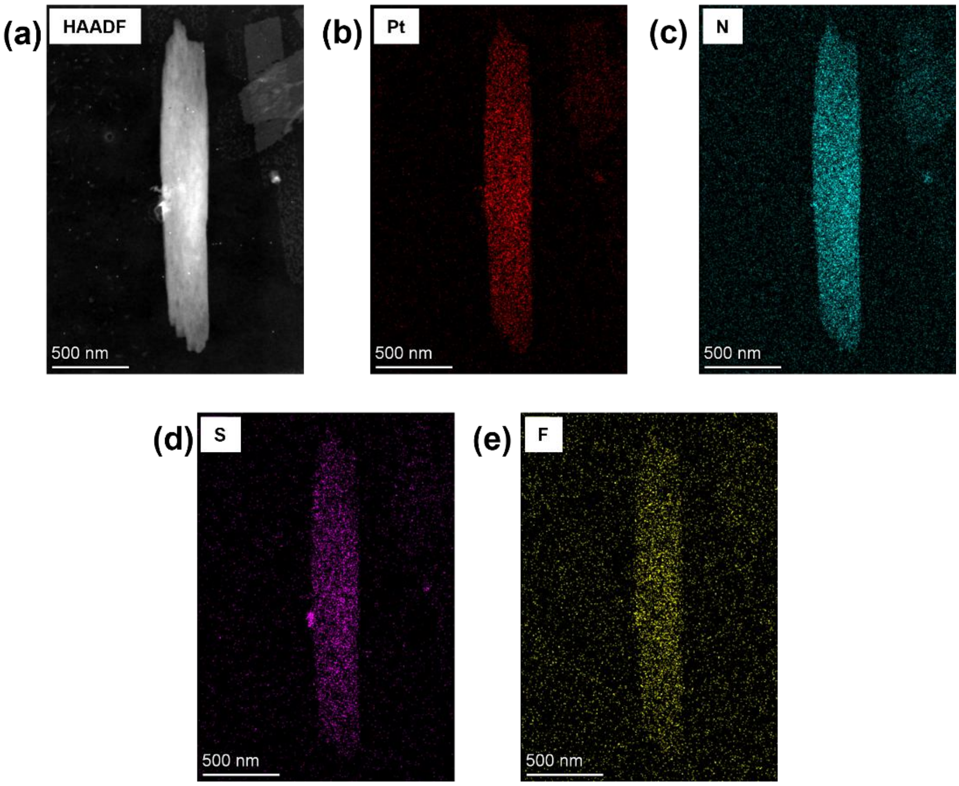

Figure S38. (a) HAADF-STEM and EDX elemental mapping images of (b) Pt, (c) N, (d) S and (e) F of **2** in acetonitrile–ethanol mixture (4:1, v/v) ([Pt] ~ 10^{-4} M) after cooling.

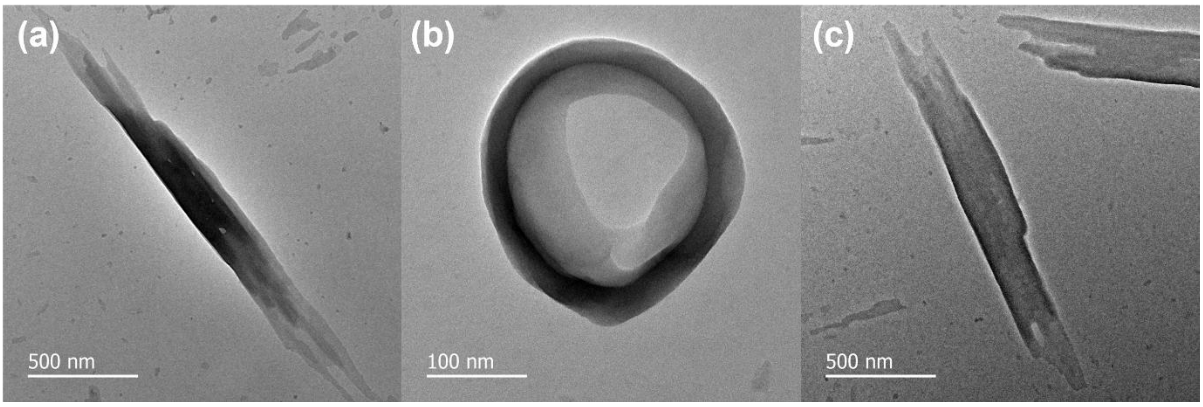

Figure S39. High-resolution TEM images of **2** in acetonitrile–ethanol mixture (4:1, v/v) ([Pt] ~ 10^{-4} M) (a) without and (b) with heat treatment, and (c) after cooling.

References

1. Sheldrick, G. M. Crystal Structure Refinement with SHELXL. *Acta Crystallogr., Sect. C:Struct. Chem.* **2015**, *71*, 3.
2. Dolomanov, O. V.; Bourhis, L. J.; Gildea, R. J.; Howard, J. A. K.; Puschmann, H. OLEX2: A Complete Structure Solution, Refinement and Analysis Program. *J. Appl. Crystallogr.* **2009**, *42*, 339.
3. Spek, A. L. PLATON SQUEEZE: A Tool for the Calculation of the Disordered Solvent Contribution to the Calculated Structure Factors. *Acta Crystallogr., Sect. C:Struct. Chem.* **2015**, *71*, 9.
4. Chatt, J.; Mann, F. G. 340. The Constitution of Complex Metallic Salts. Part X. Further Evidence for the Structure of Bridged Dipalladium Derivatives. *J. Chem. Soc.* **1939**, 1622.
5. Coulson, D. R.; Satek, L. C.; Grim, S. O., *Tetrakis(triphenylphosphine)palladium(0)*, in *Inorg. Synth.* 1972. p. 121.
6. Kershaw Cook, L. J.; Tuna, F.; Halcrow, M. A. Iron(II) and Cobalt(II) Complexes of Tris-Azinyl Analogues of 2,2′:6′,2′′-Terpyridine. *Dalton Trans.* **2013**, *42*, 2254.
7. Schultheiss, N.; Barnes, C. L.; Bosch, E. From Molecular Design to Supramolecular Design: Synthesis and Size-Selective Coordination Chemistry of 1,2-Bis(2′-pyrazineethynyl) Benzene. *Cryst. Growth Des.* **2003**, *3*, 573.
8. Weiss, K.; Beernink, G.; Dötz, F.; Birkner, A.; Müllen, K.; Wöll, C. H. Template-Mediated Synthesis of Polycyclic Aromatic Hydrocarbons: Cyclodehydrogenation and Planarization of a Hexaphenylbenzene Derivative at a Copper Surface. *Angew. Chem., Int. Ed.* **1999**, *38*, 3748.
9. Price, J. H.; Williamson, A. N.; Schramm, R. F.; Wayland, B. B. Palladium(II) and Platinum(II) Alkyl Sulfoxide Complexes. Examples of Sulfur-Bonded, Mixed Sulfur- and Oxygen-Bonded, and Totally Oxygen-Bonded Complexes. *Inorg. Chem.* **1972**, *11*, 1280.
10. Büchner, R.; Field, J. S.; Haines, R. J.; Cunningham, C. T.; McMillin, D. R. Luminescence Properties of Salts of the $[Pt(trpy)Cl]^{+}$ and $[Pt(trpy)(MeCN)]^{2+}$ Chromophores: Crystal Structure of $[Pt(trpy)(MeCN)](SbF_6)_2$. *Inorg. Chem.* **1997**, *36*, 3952.

11. Yam, V. W.-W.; Tang, R. P.-L.; Wong, K. M.-C.; Cheung, K.-K. Synthesis, Luminescence, Electrochemistry, and Ion-Binding Studies of Platinum(II) Terpyridyl Acetylide Complexes. *Organometallics* **2001**, *20*, 4476.
12. Wang, J.; Wolf, R. M.; Caldwell, J. W.; Kollman, P. A.; Case, D. A. Development and Testing of a General Amber Force Field. *J. Comput. Chem.* **2004**, *25*, 1157.
13. Zheng, X.; Chan, M. H.-Y.; Chan, A. K.-W.; Cao, S.; Ng, M.; Sheong, F. K.; Li, C.; Goonetilleke, E. C.; Lam, W. W. Y.; Lau, T.-C.; Huang, X.; Yam, V. W.-W. Elucidation of the Key Role of Pt···Pt Interactions in the Directional Self-Assembly of Platinum(II) Complexes. *Proc. Natl. Acad. Sci. U.S.A.* **2022**, *119*, e2116543119.
14. Gaussian 16, Revision A.03, Frisch, M. J.; Trucks, G. W.; Schlegel, H. B.; Scuseria, G. E.; Robb, M. A.; Cheeseman, J. R.; Scalmani, G.; Barone, V.; Petersson, G. A.; Nakatsuji, H.; Li, X.; Caricato, M.; Marenich, A. V.; Bloino, J.; Janesko, B. G.; Gomperts, R.; Mennucci, B.; Hratchian, H. P.; Ortiz, J. V.; Izmaylov, A. F.; Sonnenberg, J. L.; Williams; Ding, F.; Lipparini, F.; Egidi, F.; Goings, J.; Peng, B.; Petrone, A.; Henderson, T.; Ranasinghe, D.; Zakrzewski, V. G.; Gao, J.; Rega, N.; Zheng, G.; Liang, W.; Hada, M.; Ehara, M.; Toyota, K.; Fukuda, R.; Hasegawa, J.; Ishida, M.; Nakajima, T.; Honda, Y.; Kitao, O.; Nakai, H.; Vreven, T.; Throssell, K.; Montgomery Jr., J. A.; Peralta, J. E.; Ogliaro, F.; Bearpark, M. J.; Heyd, J. J.; Brothers, E. N.; Kudin, K. N.; Staroverov, V. N.; Keith, T. A.; Kobayashi, R.; Normand, J.; Raghavachari, K.; Rendell, A. P.; Burant, J. C.; Iyengar, S. S.; Tomasi, J.; Cossi, M.; Millam, J. M.; Klene, M.; Adamo, C.; Cammi, R.; Ochterski, J. W.; Martin, R. L.; Morokuma, K.; Farkas, O.; Foresman, J. B.; Fox, D. J. Gaussian, Inc., Wallingford CT, 2016
15. Perdew, J. P.; Burke, K.; Ernzerhof, M. Generalized Gradient Approximation Made Simple. *Phys. Rev. Lett.* **1996**, *77*, 3865.
16. Andrae, D.; Häußermann, U.; Dolg, M.; Stoll, H.; Preuß, H. Energy-Adjusted Ab Initio Pseudopotentials for the Second and Third row Transition Elements. *Theor. Chim. Acta* **1990**, *77*, 123.
17. Barone, V.; Cossi, M. Quantum Calculation of Molecular Energies and Energy Gradients in Solution by a Conductor Solvent Model. *J. Phys. Chem. A* **1998**, *102*, 1995.
18. Wang, J.; Cieplak, P.; Kollman, P. A. How Well Does a Restrained Electrostatic Potential (RESP) Model Perform in Calculating Conformational Energies of Organic and Biological Molecules? *J. Comput. Chem.* **2000**, *21*, 1049.
19. Lu, T.; Chen, F. Multiwfn: A Multifunctional Wavefunction Analyzer. *J. Comput. Chem.* **2012**, *33*, 580.

20. Allen, A. E. A.; Payne, M. C.; Cole, D. J. Harmonic Force Constants for Molecular Mechanics Force Fields via Hessian Matrix Projection. *J. Chem. Theory Comput.* **2018**, *14*, 274.

21. Lu, T. Sobtop, Version 1.0 (dev3.1), http://sobereva.com/soft/Sobtop (acessed on 31 December, 2024).

22. Van Der Spoel, D.; Lindahl, E.; Hess, B.; Groenhof, G.; Mark, A. E.; Berendsen, H. J. C. GROMACS: Fast, Flexible, and Free. *J. Comput. Chem.* **2005**, *26*, 1701.
